# Synergistic Mechanisms of Selected Polyphenols in Overcoming Chemoresistance and Enhancing Chemosensitivity in Colorectal Cancer

**DOI:** 10.3390/antiox13070815

**Published:** 2024-07-07

**Authors:** Kha Wai Hon, Rakesh Naidu

**Affiliations:** Jeffrey Cheah School of Medicine and Health Sciences, Monash University Malaysia, Jalan Lagoon Selatan, Bandar Sunway 47500, Selangor Darul Ehsan, Malaysia; hon.khawai@monash.edu

**Keywords:** chemoresistance, colorectal cancer, flavonoids, polyphenols, synergistic mechanisms, signaling pathways

## Abstract

Colorectal cancer (CRC) is a leading cause of cancer deaths worldwide. Despite significant advances in medical treatment, chemotherapy as monotherapy can lead to substantial side effects and chemoresistance. This underscores the need for therapeutic approaches that are not only pharmacologically safe but also modulate multiple potent signaling pathways and sensitize cancer cells to overcome resistance to standard drugs. In recent years, scientists have been searching for natural compounds that can be used as chemosensitizers in addition to conventional medications for the synergistic treatment of CRC. Polyphenols represent a diverse group of natural compounds that can target multiple signaling pathways in cancer cells to induce anti-cancer effects. Additionally, polyphenols have been shown to work synergistically with chemotherapeutics and other natural compounds in cancer cells. This review aims to provide a comprehensive insight into the synergistic mechanisms of selected polyphenols as chemosensitizers in CRC cells. Further research and clinical trials are warranted to fully harness the synergistic mechanisms of selected polyphenols combined with chemotherapy or natural compounds in improving cancer treatment outcomes.

## 1. Introduction

Colorectal cancer (CRC) remains one of the most prevalent and lethal malignancies worldwide, posing a significant challenge to public health systems [1]. Chemotherapy is a cornerstone treatment for CRC, often used in conjunction with surgery and radiation. The choice of chemotherapy drugs depends on the cancer stage, location, and individual patient factors. Some of the common chemotherapy drugs used to treat CRC are namely 5-fluorouracil (5-FU), irinotecan, and oxaliplatin. 5-FU is the most commonly used chemotherapeutic as the first-line therapy in CRC patients [2]. The primary function of 5-FU is to suppress nucleotide synthetic enzyme thymidylate synthase (TS) and incorporate TS metabolites into RNA and DNA, which can disrupt cell proliferation and induce apoptosis [3]. 5-FU can be used as single agent or in combination with other chemotherapeutics, such as irinotecan and oxaliplatin [2]. 5-FU, irinotecan and oxaliplatin are standard therapeutic agents of first-line treatments for metastatic colorectal cancer (mCRC) [4]. Oxaliplatin is a platinum-based chemotherapeutic that functions as an alkylating agent and inhibits DNA synthesis with the formation of inter- and intra-strand crosslinks to avoid DNA replication and transcription, leading to apoptosis [5]. Irinotecan inhibits topoisomerase I, an enzyme that relieves torsional strain in DNA by inducing single-strand breaks. Irinotecan stabilizes the complex between topoisomerase I and DNA, preventing the ligation of these single-strand breaks [6]. These drugs target cancer cells by interfering with various stages of DNA synthesis and function, which are critical for cell division and growth. The goal of chemotherapy is to improve survival rates and quality of life by reducing tumor size, slowing cancer progression, and alleviating symptoms. However, these chemotherapy drugs can also affect rapidly dividing normal cells, leading to side effects, including fatigue, nausea, and increased risk of infection.

More importantly, the development of drug resistance continues to be a major obstacle in the management of CRC, often leading to treatment failure and disease recurrence [7]. It has been estimated that the response rate of CRC patients to the abovementioned chemotherapeutics is between 40 and 50%, and this issue limits the efficiency of chemo agents, possibly leading to acquired chemoresistance when tumors become aggressive and metastatic [8]. Studies indicate that the number of publications and research interest in drug resistance in CRC has been increasing annually, highlighting its importance in the field of oncology [9]. Drug resistance significantly reduces the effectiveness of treatments like chemotherapy, targeted therapy, and immunotherapy [10]. This resistance can be intrinsic (patients are initially insensitive to drugs) or acquired (patients become insensitive after the treatment period) [11]. Moreover, approximately 50% of human cancers, including CRC, express P-glycoprotein (P-gp) at levels sufficient to confer drug resistance [12]. P-gp is a well-known efflux transporter that can pump out anticancer drugs from cancer cells, reducing drug efficacy [12]. Drug resistance remains a predominant challenge in CRC treatment, with ongoing research aiming to understand better and overcome these mechanisms to improve patient outcomes. In this context, exploring natural compounds, particularly polyphenols, has emerged as a promising avenue for overcoming drug resistance in CRC.

Polyphenols are a diverse group of naturally occurring compounds found abundantly in fruits, vegetables, tea, wine, and other plant-based foods [13]. These natural compounds are known for their antioxidant, anti-inflammatory, and anticancer properties [14]. Polyphenols can be classified into several major classes, including flavonoids, phenolic acids, and non-flavonoids (lignans, tannins, stilbenes, and others) [15]. Among all, flavonoids are the most abundant, accounting for 60% of all polyphenols [16]. Flavonoids have a basic structure of two aromatic rings linked by a three-carbon chain, which can be further divided into six different subgroups, namely flavonols, isoflavones, flavanones, flavanols, anthocyanins, and chalcones [16]. Examples of flavonoids include catechin, epigallocatechin gallate (EGCG), genistein, kaempferol, and quercetin [17]. Stilbenes are compounds that have two phenyl rings connected by an ethylene bridge [17]. For example, resveratrol is the most studied stilbene in cancer research [18]. Lignans are compounds that have two phenylpropanoid units linked by a carbon–carbon bond. Tannins are complex and high-molecular-weight polyphenols that can bind to proteins and other macromolecules [17]. Phenolic acids are simple compounds that have one or more hydroxyl groups attached to a benzoic or cinnamic acid core [17]. There are also other non-flavonoid polyphenols being studied in cancer cells. For instance, curcumin belongs to the class of polyphenols called curcuminoids, which are a subgroup of non-flavonoid polyphenols that have a diketone structure with two phenyl rings [19]. Studies have shown that polyphenols can modulate various biological pathways involved in cancer progression and drug resistance, making them potential adjuvants in CRC therapy.

The mechanisms by which polyphenols exert their effects on drug-resistant CRC cells are multifaceted. They can increase drug uptake by tumor cells, decrease drug metabolism by enzymes such as cytochromes and glutathione-S-transferases, and reduce drug efflux, which is often mediated by overexpressed transport proteins like P-glycoprotein (P-gp) [20,21]. By modulating these processes, polyphenols enhance the sensitivity of cancer cells to chemotherapeutic agents. Furthermore, polyphenols have been found to influence cell death pathways, including apoptosis and autophagy, which are often dysregulated in drug-resistant cancer cells [22,23]. They can also affect epithelial–mesenchymal transition (EMT), a process associated with increased invasiveness and resistance [24,25,26]. The modulation of reactive oxygen species (ROS) levels and DNA repair processes by polyphenols further contributes to their potential to reverse drug resistance [17,27,28]. Cancer stem cells (CSCs) are another target of polyphenols in CRC [29]. CSCs are a subpopulation of tumor cells with the ability to self-renew and differentiate, playing a crucial role in chemoresistance [30,31]. Polyphenols can disrupt the signaling pathways that maintain the stemness of CSCs, thereby reducing their contribution to drug resistance [29,32].

Epigenetic modifications, including changes in DNA methylation and histone acetylation, are involved in the development of drug resistance [33,34,35]. Polyphenols can reverse these epigenetic changes, thereby restoring the sensitivity of cancer cells to drugs [36,37,38]. Additionally, the regulation of microRNAs (miRNAs) by polyphenols represents another layer of control over gene expression related to drug resistance [39,40,41,42]. The interaction of polyphenols with the tumor microenvironment also plays a role in their therapeutic effects. They can modulate the immune response and inflammation within the tumor microenvironment, which are critical factors in the development of drug resistance [25,43,44]. Moreover, the influence of polyphenols on gut microbiota has implications for CRC treatment, as the microbiota can affect drug metabolism and the immune response to tumors [32,45,46,47,48,49]. The integration of polyphenols into CRC treatment regimens holds promise for restoring chemosensitivity, potentially revolutionizing personalized cancer therapy and improving patient outcomes. Clinical studies and trials are essential to validate the efficacy of polyphenols in overcoming drug resistance in CRC [50,51]. The bioavailability, optimal dosing, and potential side effects of polyphenols must be thoroughly investigated to translate their benefits from bench to bedside. This review aims to provide a comprehensive overview of the current understanding of the synergistic roles of selected polyphenols (curcumin, EGCG, resveratrol, quercetin, apigenin, luteolin, kaempferol, and genistein) in overcoming chemoresistance and promoting drug sensitivity in CRC. We will delve into the molecular mechanisms involved, the preclinical and clinical evidence supporting their use, and the challenges and future directions in this field. By harnessing the synergistic effects of these selected natural compounds, there is a huge potential to improve the outcomes for patients with CRC, turning the tide against drug resistance and paving the way for more effective and sustainable cancer therapies.

## 2. Curcumin

Curcumin is an active component of turmeric, a spice that is widely used in Asian cuisine and traditional medicine [52]. Curcumin is one of the most well-studied polyphenols that have multiple bioactive properties, such as anti-cancer, antibacterial, antioxidant and anti-inflammatory properties [37,53,54,55]. Curcumin has been extensively studied for its potential to modulate drug resistance in CRC via several key mechanisms. Curcumin can induce apoptosis in CRC cells by modulating apoptotic proteins, such as increasing pro-apoptotic proteins like Bax and decreasing anti-apoptotic proteins like Bcl-2 [56,57,58]. Curcumin also activates caspases, which are essential for the execution of apoptosis [59]. Curcumin also modulates the metabolism of drugs used in CRC treatment by reducing the enzymatic activity of glutathione S-transferase, which is involved in drug detoxification [60]. Curcumin also suppresses P-glycoprotein to reduce the efflux of chemotherapeutic drugs, thus increasing the intracellular concentration of these drugs [61]. Additionally, curcumin has been reported to reverse the drug resistance in CRC cells by regulating miRNA expression and enhancing sensitivity to chemotherapy, potentially improving treatment efficacy with better prognosis [62]. More importantly, accumulating evidence has highlighted on the multifaceted role of curcumin to work synergistically with other therapeutic agents and enhance chemosensitivity in CRC cells [63,64,65].

Multiple studies demonstrate that curcumin can restore chemosensitivity to 5-FU and work synergistically to improve treatment effects in drug-resistant CRC cells. Shakibaei et al. demonstrated that curcumin exhibited chemo-preventive and chemo-sensitization properties to 5-FU in mismatch repair-deficient (MMR-deficient) CRC cells in two different experimental conditions: monolayer cultures and three-dimensional high-density cultures [64,66]. Deficiencies in the MMR system is associated with genetic instability and high susceptibility to cancers including CRC [67,68,69]. The inactivation of the MMR gene can cause microsatellite instability (MSI) in CRC patients and possibly reduce the treatment response to 5-FU-based chemotherapy [70,71,72]. In a monolayer culture experiment, the combination of curcumin and 5-FU significantly reduced the cell viability of MMR-deficient HCT116 cells at lower concentrations compared to the single treatment of curcumin or 5-FU [66]. Pre-treatment with curcumin also sensitized HCT116 cells to 5-FU-induced apoptosis by promoting the release of cytochrome C from mitochondria and upregulating the downstream pro-apoptotic proteins, namely caspase-8, caspase-9, caspase-3, Bax, and PARP [66]. Curcumin also suppressed the activation of NF-κB and PI-3K/Src signaling pathways in HCT116 cells, which could be associated with the pro-survival signaling response of CRC cells to 5-FU [66]. Furthermore, the combination of curcumin and 5-FU significantly reduced proliferation and colonosphere formation while enhancing cellular apoptosis in 5-FU-resistant HCT116 cells [64]. The combination treatment of 5-FU/curcumin also significantly downregulated colon stem cell markers (CD133, CD44, and ALDH1) in parental and drug-resistant HCT116 cells [64]. As the three-dimensional high-density environment in colonosphere resembles the intercellular interactions of curcumin and 5-FU under in vivo situation, the combination of curcumin/5-FU demonstrated strong chemo-sensitizing and synergistic effects in MMR-deficient CRC cells, which could potentially increase treatment response among CRC patients.

Curcumin can mediate chemo-sensitization to 5-FU in CRC cells through the miRNA-induced suppression of EMT [26]. The combination of curcumin and 5-FU significantly suppressed proliferation, enhanced apoptosis and reduced colony formation in 5-FU-resistant HCT116 and SW480 cells, implicating the synergistic therapeutic effects in CRC cells [26]. Curcumin also upregulated EMT-suppressive miRNAs (miR-200b, miR-200c, miR-141, miR-429, and miR-101), resulting in chemo-sensitization to 5-FU as validated in a xenograft mouse model [26]. Another study by Lu et al. also reported that a high dose of curcumin suppressed EMT progression in 5-FU-resistant HCT-116 cells via the regulation of the TET1-NKD-Wnt signaling pathway [73]. Curcumin enhanced the growth inhibition, cell cycle arrest, and apoptosis in 5-FU-resistant CRC cells, promoting the expression levels of TET1 and NKD2 to inhibit the Wnt signaling pathway and reverse EMT progression [73]. Curcumin also synergized with 5-FU to induce apoptosis in 5-FU-resistant HCT-8 colon cancer cells by downregulating heat shock protein 27 (HSP27) and P-gp, which were associated with the significant effect of reversing drug resistance [74].

Noratto et al. also investigated the effects of curcuminoid in 5-FU-resistant SW480 cells [75]. Curcuminoids refers to the group of natural polyphenols that are the main active components in turmeric, including curcumin, demethoxycurcumin, and bisdemethoxycurcumin [76]. Curcuminoids significantly suppressed cell proliferation in drug-resistant CRC cells and downregulated multidrug resistance protein (MDR1) to enhance the efficacy of 5-FU [75]. Further investigation showed that curcuminoids inhibited multiple targets, namely prooncogenic specificity protein (Sp) transcription factors (Sp1, Sp3, and Sp4), Sp-regulated genes, and miR-27a [75]. Curcuminoids also induced ROS production to promote the expression of ZBTB10, which is an mRNA target of miR-27a and a transcriptional repressor of Sp expression, resulting in the inhibition of MDR1 [75]. Hence, curcuminoids re-sensitized drug-resistant CRC cells to 5-FU by promoting ROS production to disrupt the miR-27a-ZBTB10-Sp-MDR1 axis [75]. On the other hand, Howell et al. reported that curcumin enhanced the efficacy of oxaliplatin in drug-resistant HCT116 cells regardless of the p53 status [77]. The combination of curcumin and oxaliplatin significantly decreased cellular proliferation and increased caspase-3 cleavage in HCT116 cells in both in vitro and xenograft models [77]. Although curcumin did not synergise the DNA-platinating effect of oxaliplatin, the authors concluded that curcumin could demonstrate a chemo-sensitizing effect on oxaliplatin in CRC cells [77]. Other studies also demonstrated that curcumin could reverse oxaliplatin resistance in CRC cells via multiple signaling pathways. NF-κB is a transcription factor that can regulate gene expression in multiple aspects of CRC development and progression, such as apoptosis, angiogenesis, inflammation, immune response, metastasis, and proliferation [78,79].

The abnormal activation of NF-κB signaling is also associated with the upregulation of survival cues and drug resistance in CRC cells [80,81]. The combination of curcumin and oxaliplatin showed synergistic effects in reducing the proliferation of drug-resistant CRC cells [82]. The synergism of curcumin and oxaliplatin significantly suppressed CXCL8 (Interleukin-8), CXCL1 (Gro-α), and CXCL2 (Gro-β) in resistant CRC cells, resulting in the inhibition of the Akt/NF-κB signaling cascade [82]. On the other hand, transforming growth factor-beta (TGF-β) signaling is mostly involved in the tissue maintenance, inflammation, and tumorigenesis of CRC cancer cells by modulating multiple cellular processes, including growth, differentiation, apoptosis, and homeostasis [83]. Curcumin was reported to overcome oxaliplatin resistance in HCT116 colon cancer cells by modulating the TGF-β/Smad2/3 signaling pathway [84]. In the study by Yin et al., the combination of 4 μM oxaliplatin with 8 μM curcumin significantly inhibited cell growth and pro-survival signaling (phosphorylated-p65 protein and Bcl-2) with the upregulation of activated caspase-3 in the resistant cells [84]. Curcumin (alone or in combination with oxaliplatin) also inhibited EMT in resistant cells by downregulating TGF-β downstream proteins, namely Smad2, Smad3, and N-cadherin [84]. Similarly, in vivo studies also showed that curcumin significantly sensitized a CRC xenograft to oxaliplatin by suppressing Smad2/3 protein expression [84].

Cisplatin is another platinum-based chemotherapeutic widely used in the treatment of various solid tumors, such as breast, lung, and ovarian [85]. However, the effectiveness of cisplatin in CRC therapy is often unsatisfactory and associated with dose-limiting effects and drug resistance [86]. Curcumin alone or in combination with cisplatin has been shown to target non-coding RNAs (miRNAs and lncRNAs) to induce chemo-sensitization towards cisplatin treatment in CRC cells. Curcumin suppressed lncRNA KCNQ1OT1, which was overexpressed in cisplatin-resistant HCT-8 colon cancer cells [87]. LncRNA KCNQ1OT1 was shown to suppress miR-497 to promote Bcl-2 expression, which could be associated with cisplatin resistance in HCT-8 cells. Curcumin restored chemosensitivity to cisplatin in drug-resistant CRC cells by downregulating lncRNA KCNQ1OT1 and releasing miR-497 [87]. Curcumin also upregulated miR-137 to inhibit the mRNA expression of glutaminase, which was overexpressed in cisplatin-resistant HT-29 colon cancer cells [63]. The inhibition of glutaminase led to reduced glutamine metabolism and was associated with the sensitization of cisplatin-resistant CRC cells [63]. These data suggest that curcumin synergizes with cisplatin to inhibit the proliferation, glutamine metabolism and drug resistance of colon cancer cells by targeting the miR-137-glutaminase axis [63].

Several studies demonstrate that curcumin is an ideal chemo-sensitizer to reverse irinotecan resistance in CRC cells. Huang et al. reported that curcumin enhanced the cytotoxicity of irinotecan in LoVo and HT-29 colon cells by decreasing cell viability and proliferation via the synergistic effect of these compounds [88]. The combination of curcumin and irinotecan also induced cell cycle arrest and cellular apoptosis, which were associated with the increased ROS production and activation of the endoplasmic reticulum (ER) stress pathway [88]. In another study by Su et al., curcumin also significantly diminished irinotecan resistance in chemo-resistant LoVo colon cancer cells by targeting the cancer stem cell (CSC) subpopulation to induce apoptosis [89]. Additionally, curcumin was reported to attenuate irinotecan resistance in colon cancer cells by modulating EMT [90]. Zhang et al. reported that irinotecan-resistant LoVo cells showed the dysregulation of EMT markers (E-cadherin downregulated; vimentin and N-cadherin expressions upregulated), suggesting that EMT was associated with the development of drug resistance in CRC cells [90]. Curcumin significantly inhibited the proliferation of irinotecan-resistant cells, in which the combination of curcumin and irinotecan significantly promoted E-cadherin with the concurrent suppression of vimentin and N-cadherin [90].

Meanwhile, doxorubicin is another chemotherapeutic drug of the anthracycline class, which inhibits topoisomerase II to affect DNA replication and repair [91]. The long-term effectiveness of doxorubin in CRC treatment also encounters drug resistance [91]. There have been efforts to overcome the resistance of CRC cells to doxorubicin by combining it with potential multidrug resistance-reversing agents, including curcumin [92,93,94]. Previous studies showed that curcumin is a potential chemo-sensitizer to doxorubicin treatment in CRC. Curcumin attenuated drug resistance to doxorubicin in HT29 colon cancer cells by interacting with C1QBP [93]. C1QBP is a mitochondrial matrix protein that protects the mitochondria from oxidative stress by inhibiting the membrane permeability transition (MPT) pore and sustaining ATP synthesis. Interestingly, C1QBP was upregulated in doxorubicin-resistant CRC cells and associated with the acquired drug resistance [93]. Curcumin directly inhibited C1QBP to reduce cell proliferation, which could be regulated through the intracellular mechanism involving Cox-2 and P-gp [93]. Additionally, curcumin also reversed doxorubicin resistance in CRC cells by suppressing the ATP-dependent transport activity of P-gp via metabolic pathways [94]. Curcumin significantly reduced the expression of ornithine decarboxylase (ODC) associated with the downregulation of spermine and spermidine biosynthesis as well as the inhibition of D-glutamine metabolism. Subsequently, anti-oxidative stress ability and P-gp transport activity were significantly suppressed, resulting in chemo-sensitization to doxorubicin in CRC cells [94].

Most of the cell line models used for the investigation of curcumin in drug-resistant CRC involved only a single type of drug resistance, such as 5-FU, cisplatin, irinotecan, and oxaliplatin. However, chemotherapy in clinical settings often includes drug combinations, such as FOLFOX (5-FU, leucovorin, and oxaliplatin) and FOLFIRI (5-FU, irinotecan, and leucovorin), to maximize treatment efficacy and enhance patients’ prognoses [5,8]. The data of curcumin in the cell line model with multiple drug resistance is still lacking. Patel et al. successfully developed FOLFOX-resistant HCT-116 and HT-29 cells that showed an elevated expression of insulin-like growth factor-1 receptor (IGF-1R) [95]. The overexpression of IGF-1R has been associated with the development and progression of CRC, which has been significantly correlated with worse survival in CRC patients [96,97]. Curcumin was reported to synergize with FOLFOX to induce growth inhibition on chemo-resistant CRC cells, which were associated with the reduced activation of EGFR, HER-2, IGF-1R, and AKT, as well as the downregulation of COX-2 and cyclin-D [95]. Their findings suggested that curcumin targeted multiple signaling molecules to attenuate FOLFOX resistance in CRC cells, making it a potential chemo-sensitizer to improve the treatment response towards chemotherapy.

Dasatinib is a tyrosine kinase inhibitor that targets multiple kinases at nanomolar concentrations, including BCR-ABL, the Src family of kinases (Src, LCK, YES, FYN), c-KIT, EPHA2, and PDGFRβ1 in cancer cells [98,99]. In chronic myeloid leukemia (CML), dasatinib inhibits the deregulated tyrosine kinase activity of BCR-ABL, which is crucial for the growth and proliferation of leukemia cells [100,101]. In CRC, dasatinib has shown significant anti-proliferative activity in a subset of CRC cell lines, particularly those with increased Src expression at baseline, but its effectiveness as a single treatment in metastatic CRC is limited [102,103]. Ongoing research is exploring its use in combination with other treatments [104,105,106]. The combination of curcumin and dasatinib demonstrated synergistic effects on the suppression of growth factor receptors (EGFR and IGF-1R) and non-receptor (c-Src) signaling to induce growth inhibition on multiple CRC cell lines [107]. Subsequently, curcumin and dasatinib downregulated the downstream effectors and NF-κB activity, resulting in the inhibition of cell proliferation, invasion, and colonosphere formation in CRC cells [107]. The synergistic effect of curcumin and dasatinib also significantly reduced CSC population as shown by the downregulation of CSC-specific markers (ALDH, CD44, CD133, and CD166) in FOLFOX-resistant CRC cells [65].

**Figure 1:**
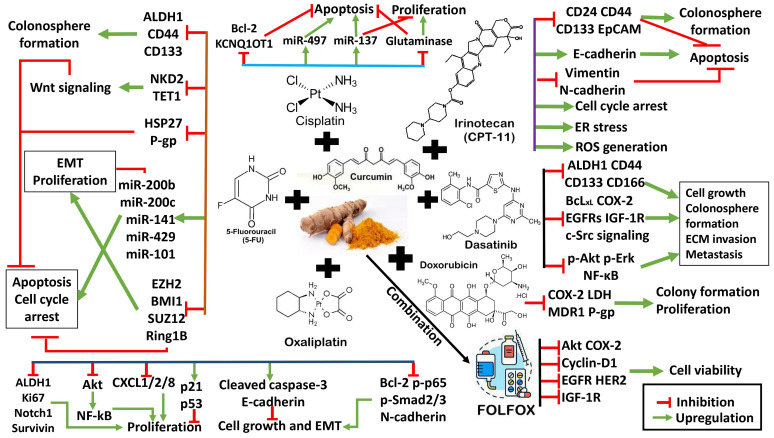
The synergistic mechanisms between curcumin and chemotherapy drugs/targeted therapies to induce chemo-sensitization in CRC cells. The red symbol represents inhibition, and the green arrow represents upregulation.

Nevertheless, curcumin is an excellent chemo-sensitizer that enhances the effectiveness of conventional chemotherapy and targeted therapies in CRC treatment, mainly by promoting apoptosis and inhibiting colonosphere formation. The addition of curcumin also reduces the required dosage of chemotherapy drugs in combination treatment compared to single agents alone, leading to less toxicity and minimal side effects. However, there are several challenges and limitations to translate the integration of curcumin and chemotherapy drugs in clinical practice. Generally, curcumin has low oral bioavailability due to poor absorption and rapid metabolism [108]. Thus, researchers explore various strategies like combining it with piperine or using nanoparticle formulations to improve delivery [109,110]. Multiple curcumin formulations have been reported in CRC preclinical and animal studies, with a wide range of IC_50_ values [23,77,111,112,113]. The lack of standardization between every formulation poses challenges in ensuring consistent potency across different curcumin products. Additionally, most of the data available are established on cell lines and animal studies that report micromolar and nanomolar concentrations. There is a knowledge gap in determining the optimal dosage and safety profile of curcumin in CRC patients [110]. Large-scale clinical trials are needed to establish curcumin’s efficacy as a chemosensitizer in diverse patient populations given that the heterogeneity of CRC with different mutational profiles may affect the patient’s response towards the combination of curcumin and chemotherapy. In summary, curcumin shows promise as an adjunct to chemotherapy in CRC treatment, enhancing its efficacy and potentially improving patient outcomes. The synergistic interactions between curcumin and chemotherapeutics/targeted therapies are illustrated in Figure 1.

## 3. Resveratrol

Resveratrol is a natural polyphenolic phytoalexin found in various fruits and vegetables such as peanuts, berries, and red grapes [114]. It has been shown to have different pharmacologic functions including anti-inflammation, cancer prevention, a lipid-lowering effect, and a hypoglycemic effect [14,114,115]. Abundant in vitro and in vivo studies have shown that resveratrol, in its interaction with standard drugs, is an effective chemosensitizer for CRC cells to chemotherapeutic agents [25,116,117,118,119,120,121]. Resveratrol can reverse drug resistance by modulating multiple targets and pathways, including transcription factors, EMT plasticity, proliferation, metastasis, angiogenesis, cell cycle, and apoptosis [25,119,121,122,123,124,125,126,127]. For instance, the co-administration of resveratrol has been shown to sensitize CRC cells to 5-FU, which remarkably increases 5-FU-induced cytotoxicity and mitigates unwanted adverse effects [25,42,117,118,119,128,129,130]. The combination of resveratrol and 5-FU also regulates the cell cycle progression of CRC cells to induce cell cycle arrest and growth inhibition [117,128,129,131]. The synergism between resveratrol and 5-FU also significantly inhibited the MAPK/Erk1/2 signaling cascade to induce apoptosis in DLD-1 colon cancer cells [42]. The mitogen-activated protein kinase (MAPK) signaling pathway is mostly associated with tumor progression, invasion, metastasis, and drug resistance in CRC cells [132]. Additionally, resveratrol also promoted miR-34a to suppress its target gene E2F3 and downstream Sirt1, resulting in growth inhibition and apoptotic induction via the miR-34a/E2F3/Sirt1 cascade [42]. Sirt1 (Sirtuin 1) is a histone deacetylase (HDAC) that controls the levels of histone acetylation and modulates gene expression, playing an important role in tumorigenesis [133]. Previously, Sirt1 overexpression was associated with stage I/II/III tumor and poor prognosis in CRC patients [133]. Interestingly, Sirt1 could have dual functions as a tumor promoter and a tumor suppressor at different stages of CRC, but Sirt1 inhibition has been reported to sensitize CRC cells to the apoptotic effect of chemotherapeutics [134].

Buhrmann et al. also demonstrated that the co-treatment of resveratrol induced chemo-sensitization to 5-FU in drug-resistant HCT116 colon cancer cells through the regulation of multiple molecular targets [25,118]. Resveratrol synergized the invasion inhibitory effects of 5-FU by inhibiting the EMT transformation of drug-resistant CRC cells via the upregulation of intercellular junctions (desmosomes, claudin-2, and E-cadherin) and downregulation of EMT factors (decreased vimentin and slug) [25]. Tumor necrosis factor-β (TNF-β) was upregulated in 5-FU-resistant HCT116 cells, which promoted chemoresistance and the development of CSCs within the resistant cells [118]. Co-treatment with resveratrol significantly suppressed the TNF-β-induced activation of NF-κB and the downstream targets (MMP-9 and CXCR4), resulting in the inhibition of cancer cell migration and CSC formation [118]. In addition, the combination of resveratrol and 5-FU also simultaneously inhibited STAT3 and Akt phosphorylation in DLD-1 colon cancer cells [117]. Deactivated STAT3 reduced the transcriptional level of human telomerase reverse transcriptase (hTERT), leading to the decrease in telomerase activity which is essential to maintain chromosomal stability during cell division [118]. Telomerase is often hyperactivated in cancers and plays an important role as a transcription co-factor in the Wnt signaling pathway to modulate cancer stemness [135]. Combined resveratrol and 5-FU also inhibited Akt phosphorylation to induce the anti-proliferation and apoptosis in CRC cells [117].

The tumor microenvironment (TME) is a complex organ-like structure that includes cancer cells, stromal cells, an extracellular matrix, and a vascular network [136]. The TME and its associated signaling play a key role to facilitate the proliferation, invasion, metastasis, and chemoresistance processes [136]. The TME can affect the sensitivity of tumor cells to apoptosis and their response to chemotherapy [136]. Recently, resveratrol has been shown to modulates chemo-sensitization to 5-FU via the β1-Integrin/HIF-1α axis in the CRC tumor microenvironment [130]. TME promoted several molecular targets in inflammation (NF-kB), vascularization (VEGF, HIF-1α), and cancer stem cell production (CD44, CD133, and ALDH1), while resveratrol significantly suppressed these factors to enhance 5-FU sensitivity in CRC cells [130]. Resveratrol could bind to β1-integrin receptors, and further studies demonstrated that resveratrol inhibited the β1-integrin/HIF-1α signaling axis in the TME to overcome the drug resistance in CRC cells [123,130,137]. Additionally, resveratrol was also reported to disrupt mitochondrial respiration in CRC cells and further amplified the cytotoxicity of oxidative stress induced by 5-FU treatment [119]. The combination of resveratrol and 5-FU can reduce inflammatory cell infiltrate, epithelial hyperplasia, and irregular crypts in rats with N-methylnitrosourea (MEN)-induced colon cancer compared to rats without treatment [138]. Resveratrol can also sensitize CRC cells to 5-FU treatment through mechanisms such as apoptosis, anti-inflammatory effects, oxidants, and the regulation of cell cycle distribution [138]. These findings suggest that resveratrol could be an important additive to 5-FU chemotherapy, potentially improving outcomes for CRC patients facing drug resistance.

Resveratrol can also induce chemo-sensitization to oxaliplatin in CRC cells. Co-treatment with resveratrol and oxaliplatin showed a significant inhibition of cell growth at a lower concentration than that of the single compounds alone [120,139]. Resveratrol upregulated miR-34c in both in vitro and in vivo studies [139]. In cell line models (HT29 and HCT116), the resveratrol-induced miR-34c upregulation suppressed its downstream target KITLG, which could be associated with the inhibition of cell proliferation, migration, and invasion [139]. The authors also observed that resveratrol showed the enhanced inhibition of miR-34c-KITLG in the presence of p53 activation, suggesting that the inhibitory mechanism could be achieved probably through inactivating the PI3K/Akt pathway [139]. In addition, resveratrol also activated the miR-34c-KITLG axis in a CRC xenograft of mice and reduced the inflammatory factor interleukin 6 (IL-6) [139]. Taken together, resveratrol demonstrated a synergistic effect with oxaliplatin in an miR-34c-dependent manner. Similarly, the combination of resveratrol and oxaliplatin showed synergistic effects in increasing different forms of cell deaths (apoptosis and primary/secondary necrosis in Caco-2 CRC cells), while the conditioned medium of co-treated cells significantly increased the release of pro-inflammatory cytokines TNF-α and IL-8 from human macrophages [120]. Hence, resveratrol synergizes with oxaliplatin to induce cytotoxicity in CRC cells, which can be further enhanced with inflammation.

Resveratrol can also synergize with doxorubicin to induce apoptosis and chemo-sensitization in drug-resistant CRC cells by downregulating the expression and activity of multidrug resistance proteins (BCRP, MRP1, and P-gp) and metabolic enzymes (GST and CYP3A4) [125,140]. Breast cancer resistance protein (BCRP/ABCG2), multidrug resistance protein 1 (MRP1/ABCC1), and P-gp are the ATP-binding cassette (ABC) transporter family members that carry out drug efflux and their overexpression contributes to the development of chemoresistance in cancer cells [141,142,143]. Glutathione S-transferase (GST) and cytochrome P450 (CYP) enzymes are well-known drug-metabolizing enzymes that can promote intracellular drug metabolism to reduce the efficacy of chemotherapy in cancer cells [144]. Furthermore, the co-administration of curcumin and resveratrol can improve anti-cancer activities and synergistic effects in CRC cells by regulating multiple molecular targets and signaling pathways [145,146]. The combination of curcumin and resveratrol significantly induced synergistic effects on the growth inhibition and apoptosis of colon cancer cells in vitro and in vivo by attenuating NF-κB activity and the activation of EGFR and IGF-1R signaling [145]. Research has shown that the combination of resveratrol and curcumin can activate pro-apoptotic genes, including *PMAIP1*, *BID*, *ZMAT3*, *CASP3*, *CASP7*, and *FAS*, in CRC cells, depending on mutational profiles (KRAS, PIK3CA, PTEN, BRAF, and TP53) [146]. Microsatellite instability (MSI) and microsatellite stability (MSS) are two different biomarkers that indicate DNA stability in cancer cells, in which MSI is generally more invasive than MSS and carries mutations in DNA mismatch repair genes [147]. The combination had a synergistic effect on the MSI CRC cell line (DLD-1) but an additive effect on the MSS CRC cell line (Caco-2) [146].

**Figure 2:**
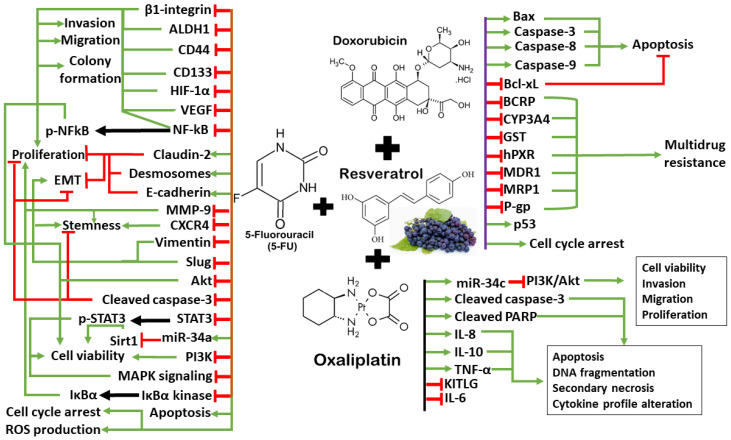
The synergistic mechanisms between resveratrol and chemotherapy drugs to induce chemo-sensitization in CRC cells. The red symbol represents inhibition, the green arrow represents upregulation, and the black arrow indicates phosphorylation or enzymatic regulation.

The synergism of resveratrol and chemotherapy drugs is promising to modulate apoptosis, DNA damage, invasion, migration, and inflammatory response in CRC subtypes of distinct mutational profiles. The ability of resveratrol to modify multiple subcellular pathways that may suppress cancer cell plasticity and the reversal of multidrug resistance are critical parameters for understanding its anti-cancer effects. However, the addition of resveratrol to chemotherapy could have adverse effects. A previous study reported that resveratrol demonstrates estrogen-like properties, which may activate gene transcription by estrogen and androgen receptors, leading to cancer cell proliferation [148]. A high dosage of resveratrol could induce gastrointestinal discomfort and diarrhea [149]. In addition, resveratrol has been reported to suppress CYP450 enzymes, affecting the levels of drugs that are metabolized by these enzymes [150]. Therefore, larger well-designed trials are necessary to determine the safety profile of resveratrol when combining it with chemotherapy to treat CRC patients. Resveratrol holds promise as a chemosensitizer to synergize with few selected cancer drugs (5-FU, doxorubicin, and oxaliplatin), while its practical application in clinical practice requires further research and optimization. The synergistic mechanisms between resveratrol and chemotherapy drugs are illustrated in Figure 2.

## 4. Epigallocatechin Gallate (EGCG)

Epigallocatechin-3-gallate (EGCG) is a major active component found in green tea that belongs to the catechin subclass under polyphenols [151]. EGCG and other related catechins can act as potent antioxidants to protect target cells against cellular damage caused by free radicals, reducing inflammation and prevent certain chronic conditions, including heart disease, diabetes, and cancers [17]. The antioxidant and anti-inflammatory effects of EGCG have garnered attention for their therapeutic potential in CRC. Research has shown that EGCG can inhibit the growth of CRC cancer stem cells (CSCs) by suppressing the Wnt/β-catenin pathway [152]. CSCs play a crucial role in cancer development and targeting them may be an effective strategy for intervention [153]. EGCG reduces spheroid formation capability, CSC marker expression, and cell proliferation while inducing apoptosis in CRC cells [152]. One study demonstrated that EGCG could suppress colorectal cancer cell growth by inducing apoptosis and downregulating STAT3, which is involved in cell growth and survival [154]. EGCG also inhibits Notch signaling to reduce proliferation, induce apoptosis and cell cycle arrest in CRC cells [155]. In addition to its cancer preventive properties, EGCG may serve as an adjunct to conventional chemotherapy in CRC.

Another study found that EGCG enhanced the chemosensitivity of 5-FU in CRC by targeting CSCs and regulating different molecular targets and miRNAs [156]. This study showed that EGCG treatment resulted in the suppression of Notch1, Bmi1, Suz12, and Ezh2 and upregulated self-renewal-suppressive miRNAs, (miR-34a, miR-145, and miR-200c), which are the key components often dysregulated in 5FU-resistant CRC cells [156]. In addition, EGCG enhances the sensitivity of CRC cells to 5-FU by inhibiting the GRP78/NF-κB/miR-155-5p/MDR1 pathway [157]. GRP78, also known as binding immunoglobulin protein (BiP) or HSPA5, is a member of the heat-shock protein 70 (HSP70) family [158]. It plays a crucial role in the folding and assembly of proteins within the endoplasmic reticulum (ER) [158]. GRP78 is involved in various cellular processes, including serving as a chaperone that assists in protein folding, ensuring the quality control of newly synthesized proteins, and regulating ER stress signaling pathway [159]. EGCG treatment in 5-FU-resistant CEC cells (DLD-1 and HCT116 cells) inhibited GRP78 expression, which activated the NF-κB pathway and the upregulation of miR-155-5p [157]. Further analysis showed that miR-155-5p significantly suppressed MDR1 gene expression, resulting in the intracellular accumulation of 5-FU in drug-resistant CRC cells [157]. Subsequently, this led to the activation of the apoptotic cascade (caspase-3 and PARP activation, Bcl-2 reduction, and Bad overexpression), suggesting that EGCG could enhance 5-FU sensitivity via the GRP78/NF-κB/miR-155-5p/MDR1 pathway in CRC cells [157].

Interestingly, another study by Wu et al. reported that EGCG enhanced the chemosensitivity of CRC cells to irinotecan through GRP78-mediated endoplasmic reticulum stress (ERS) [160]. GRP78 was overexpressed in CRC tumor specimens, and inhibiting GRP78 reduced drug sensitivity, while its overexpression increased apoptosis after drug combination [160]. Co-treatment with EGCG and irinotecan promoted intracellular GRP78 expression in RKO and HCT116 cells, while the addition of EGCG inhibited GRP78 membrane translocation induced by irinotecan [160]. EGCG increased the intracellular accumulation of GRP78 and prevented its cell membrane translocation to induce ERS in CRC cells, resulting in DNA damage and apoptosis [160]. By combining it with different types of chemotherapeutics, such as 5-FU and irinotecan, EGCG could have different regulatory mechanisms on GRP78 to induce cellular stress and the activation of pro-apoptotic signaling pathways, which ultimately lead to chemo-sensitization and apoptosis in drug-resistant CRC cells. Furthermore, EGCG and irinotecan synergistically caused more extensive DNA damage in CRC cells by inhibiting topoisomerase I, leading to S or G2 phase arrest [161]. The combined treatment also induced apoptosis by promoting autophagy, while EGCG also inhibited the tumor suppressive Hippo signaling pathway to activate the downstream YAP and induce anti-cancer effects in CRC cells [161]. Autophagy is a cellular degradation process that removes unnecessary or dysfunctional components through a lysosome-dependent regulated mechanism to maintain cell survival [162].

Autophagy can modulate CRC progression in both pro- and anti-tumorigenic ways [162]. In the early stages of CRC, autophagy inhibits tumor development by maintaining DNA stability, inducing tumor death, and enhancing immune surveillance [163]. However, as CRC progresses, autophagy may promote tumor development by enhancing tumor metabolism and other pathways [163]. Nevertheless, EGCG has been reported to synergize the therapeutic effects of cisplatin and oxaliplatin through the autophagic pathway in CRC cells [164]. The co-treatment of EGCG and cisplatin or oxaliplatin synergistically reduced cell proliferation and induced cell death in DLD-1 and HT-29 cells [164]. The combinatorial effects of EGCG plus cisplatin or oxaliplatin led to elevated autophagy in CRC cells and was associated with the accumulation of the LC3-II protein, the presence of acidic vesicular organelles (AVOs), and the formation of autophagosome [164]. These findings suggest that EGCG could be a promising agent for CRC intervention through its ability to target CSCs and enhance the effectiveness of conventional chemotherapeutic drugs. While pre-clinical studies have shown promising results, the translation of EGCG’s effects into clinical practice remains limited due to several factors. EGCG has poor bioavailability due to its rapid metabolism and low absorption [165]. This limits its effectiveness when administered orally. Strategies to enhance its bioavailability, such as encapsulation or combining it with other compounds, are being explored [165]. Furthermore, the optimal dosage of EGCG for chemo-sensitization is not well defined. Too low a dose may be ineffective, while high doses could lead to adverse effects.

**Figure 3:**
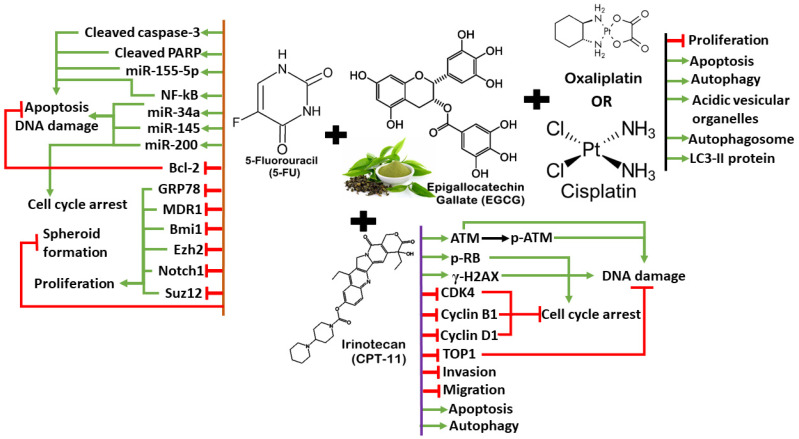
The synergistic mechanisms between EGCG and chemotherapy drugs/targeted therapy to induce chemo-sensitization in CRC cells. The red symbol represents inhibition, and the green arrow represents upregulation.

Previous studies also highlighted that the possible interaction between EGCG with certain medications and metabolic enzymes, affecting drug efficacy and safety profile in cancer treatment. For instance, EGCG could inhibit transport of irinotecan and its metabolite SN-38 into biliary elimination, resulting in the prolonged half-life of accumulated drugs and increasing the risk of systemic toxicity [166]. EGCG also inhibits CYP3A4 to affect the intracellular concentration of drugs metabolized by this enzyme, including imatinib used in leukemia and tamoxifen used in hormone receptor-positive breast cancer [167,168]. Clinicians need to consider potential drug interactions when using EGCG as an adjuvant. The ability of EGCG to synergize with some chemotherapy drugs and target multiple aspects of CRC tumorigenesis, including autophagy, apoptosis, DNA damage, cell cycle arrest, and anti-proliferation, makes it a promising candidate for CRC management, although its limitations and lack of robust clinical evidence warrant cautious use. The synergistic mechanisms between EGCG and chemotherapy drugs/targeted therapy are illustrated in Figure 3.

## 5. Quercetin

Quercetin is a polyphenolic flavonoid commonly found in fruits and vegetables, and it has been studied for its potential anti-carcinogenic properties, particularly in relation to CRC [169]. Research indicates that quercetin may impact CRC by modulating various molecular targets and signal transduction pathways, such as Wnt/β-catenin, PI3K/AKT, MAPK/Erk, JNK, p38, p53, and NF-κB [170]. Studies have shown that quercetin can promote the loss of cell viability, apoptosis, and autophagy in cancer cells through these pathways, suggesting a potential therapeutic role for quercetin in CRC treatment [170,171]. Additionally, quercetin has been reported to preferentially induce apoptosis in CRC cells harboring mutant KRAS, which is significant since KRAS mutations occur in a substantial percentage of CRC cases [172]. Quercetin has been shown to influence drug resistance in CRC through several mechanisms. Quercetin can synergize the apoptotic effects of 5FU in MSI CRC cells (CO115 and HCT116) via the regulation of tumor suppressor p53 [173]. CRC tumors with microsatellite instability (MSI) have been associated with 5-FU resistance which hinders treatment efficacy [147,174,175,176,177]. Quercetin alone and in combination with 5-FU promoted p53 expression in both cell lines [173]. Co-treatment with quercetin and 5-FU enhanced the apoptotic effect in CO115 cells via the apoptotic mitochondrial pathway (the cleavage of caspase-3, -9, and PARP as well as Bcl-2 downregulation) [173]. The knockdown of p53 by siRNA in CO115 cells and p53 knockout in HCT116 cells totally attenuated the apoptotic effect of quercetin and 5-FU, implicating that p53 was involved in the synergistic mechanism of the combined treatment in CRC cells [173].

More recently, quercetin has been shown to modulate the nuclear factor erythroid 2-related factor (Nrf2)/heme oxygenase-1 (HO-1) pathway to reverse 5-FU resistance in colon cancer cells [178]. Previous study suggests that ROS production could be due to upregulation in cancer cells due to increased mitochondrial activity and accelerated metabolism, which are required to sustain rapid cell proliferation and cancer development [179]. Tang et al. demonstrated that the combination of quercetin and 5-FU significantly inhibited oxidative stress-related factors (SOD, CAT, GPx, and GR) and ROS production in 5-FU-resistant colon cancer cells, resulting in growth inhibition and enhanced apoptosis compared to single treatments [178]. Nrf2 is a transcription factor that plays an important role in regulating oxidative stress, in which Nrf2 overexpression is associated with tumor proliferation, drug resistance, and poor prognosis [180,181,182,183,184]. Nrf2 downstream proteins, including HO-1 and SOD, are antioxidant proteins which can assist cancer cells to resist oxidative stress [185]. Quercetin and 5-FU synergistically suppressed the Nrf2/HO-1 pathway, suggesting that the regulation of cellular response towards oxidative stress is crucial for the anti-cancer effects of combined treatment to overcome 5-FU resistance in CRC cells.

The addition of quercetin to 5-FU chemotherapy also enhances apoptosis in HCT-116 and Caco-2 CRC cells compared with 5-FU alone [186]. This combination significantly inhibited miR-27a via a synergistic effect, resulting in the downregulation of Wnt/β-catenin signaling and cyclin D1 expression [186]. Thus quercetin potentiates 5-FU cytotoxic effects in CRC cells by inhibiting the miR-27a/Wnt/β-catenin signaling pathway [186]. Quercetin also synergizes the anti-cancer effects of 5-FU in HT-29 colon cancer cells to induce apoptosis associated with the upregulation of p53, Bax, p38 MAPK, and PTEN gene expression [187]. Co-treatment with quercetin and 5-FU also significantly suppressed VEGF, which is involved in angiogenesis, along with downregulating the anti-apoptotic genes (Bcl-2, mTOR, and Akt) [187]. By enhancing the apoptotic effects of 5-FU, quercetin is a potential chemo-sensitizer that can minimize the toxicity and side effects of 5-FU in CRC clinical treatment. Another study found that quercetin, when combined with oxaliplatin to treat HCT116 colon cancer cells, can synergistically inhibit glutathione reductase activity [188]. This leads to a decrease in intracellular glutathione levels, an increase in reactive oxygen species production, and a reduction in cell viability compared to treatment with oxaliplatin alone [188]. Similarly, the authors also investigated the combined effects of oxaliplatin and quercetin in an HCT116 xenograft mouse model [188]. Oxaliplatin and quercetin synergistically reduced tumor size and induced apoptosis in xenograft tumors by upregulating Bax, cytochrome C release, and PARP cleavage [188]. These outcomes demonstrate a huge potential of combining quercetin and oxaliplatin for further clinical investigation.

**Figure 4:**
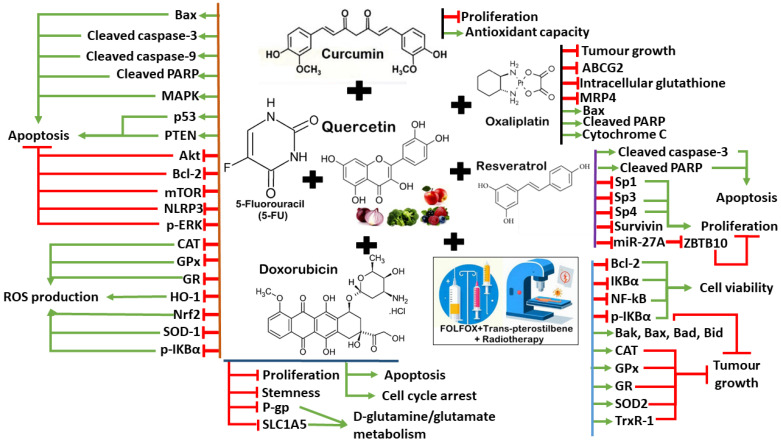
The synergistic mechanisms between quercetin and chemotherapy drugs/targeted therapies to induce chemo-sensitization in CRC cells. The red symbol represents inhibition, and the green arrow represents upregulation.

Additionally, fermented quercetin has been reported to induce cytotoxicity and cell death in 5-FU-resistant CRC cells [189]. This effect is associated with the downregulation of NLRP3 expression and ERK phosphorylation, indicating the role of NLRP3 in the development of drug resistance to 5-FU in CRC cells [189]. Quercetin can overcome colon cancer cells’ resistance to doxorubicin by inhibiting SLC1A5 which is a major glutamine transporter commonly overexpressed in cancer cells to support growth and survival [190]. Quercetin can enhance the cytotoxicity of doxorubicin in SW620/Ad300 cells by inhibiting the transport activity of P-glycoprotein and antagonize glutamine metabolism [190]. Moreover, quercetin has been reported to induce cell cycle arrest and apoptosis in the CD133+ CSC subpopulation of HT29 colon cancer cells, which also enhances the anticancer effects of doxorubicin [191]. This effect is clinically important to reduce the serious side effects associated with doxorubicin therapy at high concentrations on normal cells [191]. These findings suggest that quercetin could potentially be used to enhance the efficacy of certain chemotherapy drugs by modulating apoptosis, cancer cell metabolism, ROS production, and proliferation in CRC cells. More importantly, quercetin can also synergize with other polyphenols (curcumin and resveratrol) to induce anti-cancer effects, suggesting their huge potential to complement chemotherapy/radiotherapy in patients with CRC. Nevertheless, more research is needed to fully understand the interactions between quercetin, curcumin, and resveratrol in cancer treatment. Similarly, the safety profile and long-term effects of quercetin uptake in patients with CRC also require further investigation by looking into the bioavailability, optimal dosage and possible interaction with other medications. The synergistic mechanisms between quercetin and chemotherapy drugs/targeted therapies are illustrated in Figure 4.

## 6. Apigenin

Apigenin is a natural flavonoid that is classified under the flavone class and found in many fruits and vegetables. Apigenin has shown potential anti-cancer effects on CRC cells. Studies have indicated that apigenin can suppress cell proliferation, migration, and invasion by inhibiting the Wnt/β-catenin signaling pathway [192]. This pathway is crucial for cell growth and division, and its abnormal activation is linked to tumorigenesis [193]. Further research suggests that apigenin impacts various cellular and molecular mechanisms, potentially affecting pathways like PI3K/AKT/mTOR, MAPK/ERK, and others involved in cell cycle regulation and apoptosis [194]. Additionally, apigenin has been observed to restrict glycolysis in CRC cells by targeting specific enzymes and proteins, resulting in the inhibition of tumor progression [195]. These actions can contribute towards the anti-cancer effects of apigenin to function as a potential chemosensitizer in CRC cells. Apigenin has been reported to synergize with 5-FU and enhance apoptotic effects in 5-FU-resistant HCT116 cells [196]. Apigenin significantly suppresses thymidylate synthase (TS), which is the target protein of 5-FU. The overexpression of TS was reported in 5-FU-resistant CRC cells, which could be associated with the reduction in 5-FU sensitivity [196]. Co-treatment with 5-FU and apigenin also further induced P53 upregulation, ROS production, the dysregulation of calcium ion signaling, cell cycle arrest, and the depolarization of mitochondrial membrane potential in colon cancer cells [196]. Apigenin also inhibited forkhead box protein M (FOXM1) which is a transcription factor modulating 5-FU sensitivity in CRC cells [196]. Apigenin can restore the chemo-sensitization of CRC cells towards 5-FU by suppressing TS, while the apoptotic effects of combined 5-FU and apigenin are modulated via the functional P53 [196]. Further studies are essential to determine the molecular interaction of the FOXM1–TS axis when regulated by combined 5-FU and apigenin treatment to induce apoptosis in CRC cells.

In addition, Sen et al. developed a liposomal nanocarrier for the simultaneous delivery of apigenin and 5-FU into HCT-15 and HT-29 CRC cells [197]. This dual-drug-loaded liposomal formulation significantly affected the synergistic therapeutic efficacy of apigenin and 5-FU in reducing cell proliferation and angiogenesis associated with the upregulation of apoptotic potential [197]. The dual-drug liposome also promoted AMPK activation and COX-2 downregulation in CRC cells, which could be associated with the attenuation of the Warburg effect to reduce tumor proliferation [197]. The Warburg effect generally refers to metabolic changes, such as increased glucose uptake and lactate production, in cancer cells to support tumor proliferation and survival [198]. The dual-drug-loaded liposome also improved in vivo tumor regression in an HT-29 nude mice xenograft model [197]. On the other hand, co-treatment with apigenin increased the potency of irinotecan by four times in HT-29 and HRT-18 CRC cells [199]. Apigenin and irinotecan synergistically promote the expression of anti-metastatic protein CD26 on cancer cell surfaces, which may limit local signals that promote tumor progression [199]. CD26 activates its downstream enzymes, namely dipeptidyl peptidase (DPPIV) and ecto-adenosine deaminase (eADA), to suppress tumor metastasis [199]. Thus, apigenin could enhance the beneficial effects of irinotecan in CRC cells independent of its direct cytotoxic action [199]. Furthermore, apigenin has been reported to induce autophagy and apoptosis in cisplatin-resistant HT-29 colon cancer cells [200]. Apigenin could suppress the m-TOR/PI3K/Akt signaling pathway to induce its anti-cancer potential in drug-resistant colon cancer cells, suggesting that apigenin could be a potential chemo-sensitizer to cisplatin in CRC cells [200].

**Figure 5:**
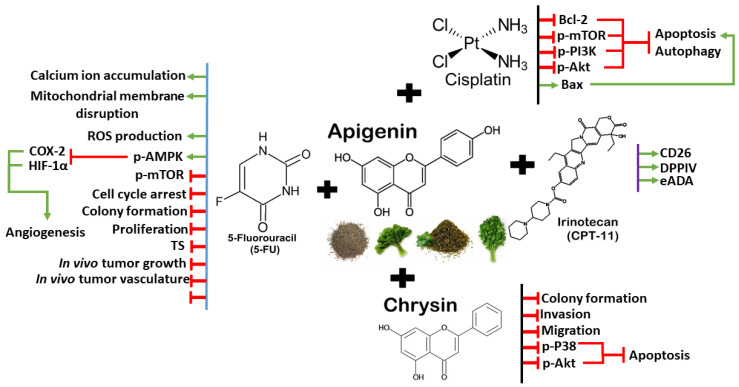
The synergistic mechanisms between apigenin and chemotherapy drugs/targeted therapy/another polyphenol to induce chemo-sensitization in CRC cells. The red symbol represents inhibition, and the green arrow represents upregulation.

Combination therapy involving apigenin has shown promising outcomes in overcoming drug resistance and boosting anti-cancer properties. Apigenin sensitizes chemo drugs through various pathways, including reducing overexpressed genes, inhibiting specific proteins (such as Akt, PI3K, and mTOR), and promoting apoptosis in treated cells. These co-therapies could help to reduce the toxicity of chemotherapeutic agents and potentially lead to the development of drugs with a high therapeutic index. Compared to popular polyphenols, namely curcumin, EGCG, resveratrol, and quercetin, the development of apigenin as a chemo-sensitizer is still in its infancy and its specificity for cancer cells needs further investigation. Animal studies and clinical trials are necessary to validate its safety profile and efficacy for clinical practice. The synergistic mechanisms between apigenin and chemotherapy drugs/targeted therapy/another polyphenol are illustrated in Figure 5.

## 7. Luteolin

Luteolin is a bioflavonoid with potential anti-cancer properties, and it has been studied for its therapeutic effects in CRC [201]. Luteolin and quercetin are quite similar in chemical structure, which is based on a 15-carbon skeleton with a chromone core comprising bicyclic 1,4-benzopyrone (A- and C-rings) substituted on carbon 2 with a catechol moiety (B-ring) [202]. Ring A features a phloroglucinol substitution pattern with two free hydroxyl groups in position 5 and 7 [202]. Quercetin differs from luteolin by only one additional hydroxyl group in position 3 [202]. Research suggests that luteolin can suppress the growth and migration of colon cancer cells by inhibiting the IL-6/STAT3 signaling pathway [203]. This pathway is involved in inflammation and cancer progression, and its inhibition may reduce the proliferation rate and invasion ability of cancer cells [204]. Another study found that luteolin could suppress CRC cell metastasis by regulating the miR-384/pleiotrophin axis [205]. Pleiotrophin is a protein associated with cancer progression, and luteolin’s ability to modulate its expression through miRNA could be a promising approach for cancer therapy [205]. Additionally, luteolin can reduce oxidative stress to modulate cancer development, and it may modify the Wnt/β-catenin signaling pathway, which is often deregulated during neoplastic development [201,206,207]. It also impacts DNA repair by modulating the MAPK pathway, which could prevent tumorigenesis in CRC [208]. More importantly, increasing evidence demonstrates that luteolin is a potential compound that can synergize with the anti-cancer effects of oxaliplatin in CRC cells. Co-treatment with luteolin enhances the apoptotic effect of oxaliplatin in drug-resistant CRC cells via several mechanisms.

Nuclear factor E2-related factor 2 (Nrf2) is a transcription factor that regulates cellular antioxidants and detoxifying response, while the aberrant activation of Nrf2 has been associated with chemoresistance in cancer cells [180,183,184,209,210]. Interestingly, previous studies suggest that luteolin could have the opposite regulation of Nrf2 and its downstream pathway to induce anti-cancer effects in drug-resistant CRC cells. Chian et al. reported that luteolin sensitized two oxaliplatin-resistant CRC cell lines (HCT116 and SW620) to chemotherapeutic drugs (oxaliplatin, cisplatin, and doxorubicin) by inhibiting the Nrf2 pathway, which may reduce drug resistance [211]. Luteolin significantly inhibited Nrf2 and its target genes [NADPH quinone oxidoreductase 1 (NQO1), heme oxygenase-1 (HO-1), and GSTα1/2] in resistant cells, while the combination of luteolin with different chemotherapeutics demonstrated synergistic effects in anti-cancer activities. However, another study by Jang et al. demonstrated that luteolin upregulated the Nrf2 downstream protein and HO-1 expression to induce apoptosis in HCT116 cells [212]. According to this study, the initial treatment using oxaliplatin induced cell cycle arrest at the G_0_/G_1_ phase in HCT116 cells, while co-treatment with luteolin induced apoptosis via Nrf2/ARE/HO-1 activation [212]. Luteolin-induced apoptosis and oxaliplatin-induced cell cycle arrest both depend on p53 function [212]. The authors proposed that luteolin-induced apoptosis may override oxaliplatin-induced cell cycle arrest by increasing HO-1 expression in p53-expressing cells [212]. Previously, luteolin was reported to promote Nrf2 expression in HT29 colon cancer cells via the action of DNA demethylase, resulting in the enhanced interaction between Nrf2 and p53 that eventually led to apoptosis [213]. Nevertheless, luteolin could enhance the anti-cancer effects of oxaliplatin in CRC cells via the p53 mechanism [212].

In addition, Jang et al. also further investigated the anti-cancer effect of luteolin alone and in combination with oxaliplatin in an HCT116 xenograft tumor [214]. Their findings showed that the combination of luteolin and oxaliplatin had synergistic effects on the growth inhibition and apoptosis of the HCT116 xenograft [214]. Functional analysis also demonstrated that luteolin could upregulate the Nrf2/ARE/HO-1 axis in HCT116 cells mainly through both AKT activation and AMPK inhibition, resulting in a significant apoptotic effect [214]. AMP-activated protein kinase (AMPK) is a well-known regulator of cellular energy homeostasis, which increases glucose and fatty acid uptake and oxidation in response to low energy levels [215]. In cancers, AMPK has dual functions as a tumor suppressor and a pro-tumorigenic, in which its tumor-promoting role is associated with drug resistance by enhancing glycolysis and metabolic reprogramming to support tumor proliferation [216,217,218]. Luteolin-induced AMPK inhibition significantly affected glycolytic metabolism in HCT116 cells, leading to energy depletion and the consequent suppression of cell proliferation [214]. Thus, luteolin can synergize the anti-cancer effects of oxaliplatin in CRC cells via AMPK inhibition and the activation of the Nrf2/ARE/HO-1 pathway. Furthermore, luteolin also enhances the chemosensitivity of SW480 colon cancer cells to oxaliplatin through the PPARγ/OCTN2 pathway [219]. Organic cation/carnitine transporter 2 (OCTN2) is a member of the solute carrier superfamily which facilitates oxaliplatin uptake in cancer cells, while peroxisome proliferator-activated receptor γ (PPARγ) binds to the PPAR-response element within the first intron of the *OCTN2* gene [220,221]. Luteolin significantly increased the expression of OCTN2 via the upregulation of PPARγ [219]. Luteolin also enhanced the binding affinity of OCTN2 toward oxaliplatin, which led to intracellular accumulation of oxaliplatin [219]. At the same time, luteolin also promoted the expression of PDZ domain-containing 1 (PDZK1) and PDZ domain-containing 3 (PDZK2), which increased OCTN2 expression on cancer cell surfaces with enhanced transporter activity [219]. Therefore, luteolin promoted the uptake and intracellular accumulation of oxaliplatin in CRC cells via the upregulation of the PPARγ/OCTN2 pathway, overcoming drug resistance. These findings highlight the versatility of luteolin and its ability to interact with various signaling pathways and proteins, making it a potential candidate to complement chemotherapy in CRC treatment.

**Figure 6:**
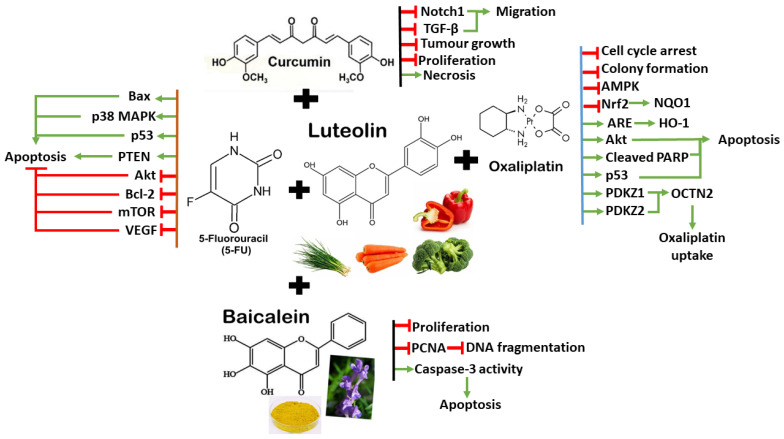
The synergistic mechanisms between apigenin and chemotherapy drugs/other polyphenols to induce chemo-sensitization in CRC cells. The red symbol represents inhibition, and the green arrow represents upregulation.

While more clinical trials are needed, these studies highlight luteolin’s potential as a complementary therapy for CRC. Unlike quercetin, current research progress on luteolin studies focus on in vitro experiments using cancer cell lines. Animal studies and clinical trials are scarce, while luteolin’s optimal dosage for anticancer effects remains unclear. Interactions between luteolin with existing chemotherapy regimens, long-term effects, potential toxicity, and side effects remain uncertain. These limitations emphasize the need for more comprehensive investigations and rigorous clinical trials to validate luteolin’s effects in actual CRC patients. The synergistic mechanisms between luteolin and chemotherapy drugs/other polyphenols are illustrated in Figure 6.

## 8. Kaempferol

Kaempferol belongs to the flavonol subclass of flavonoids and can be found in various fruits, vegetables, and medicinal plants [222]. Kaempferol has emerged as a promising compound in cancer therapy as it demonstrates multifaceted effects on cancer cells and tumor microenvironments, making it a subject of intense research in recent decades [223,224,225,226]. Kaempferol has demonstrated potent anti-cancer properties in both experimental and biological studies. It interferes with various signaling pathways within cancer cells, leading to growth inhibition and apoptosis in different tumor types [227]. By disrupting aberrant cellular processes, kaempferol acts as a guardian against uncontrolled cell proliferation. At the molecular level, kaempferol modulates key elements in cellular signal transduction pathways linked to apoptosis, angiogenesis, inflammation, and metastasis. Kaempferol enhances the body’s antioxidant defense against free radicals, which play a role in cancer development.

Kaempferol also shares structural similarities with the estrogen hormone. As a flavonoid, kaempferol possesses a ring structure that bears resemblance to the steroidal backbone found in estrogen [228]. Kaempferol is classified as a phytoestrogen, meaning it mimics estrogenic effects to some extent [222]. While kaempferol is not a true estrogen, its structural resemblance allows it to interact with estrogen receptors (ERs) in the body [222]. Kaempferol can bind to ERs, albeit with lower affinity than endogenous estrogens [222]. This interaction may influence gene expression and cellular responses. Some studies suggest that kaempferol may have protective effects against breast cancer by modulating the ER signaling pathways [224,228]. In CRC, combining kaempferol with 5-FU has shown promising results in the greater inhibition of CRC cell viability compared to using either agent alone [229,230,231]. The growth inhibition of the combined treatment is associated with reduced proliferation ability and increased apoptosis [229,230,231]. Kaempferol and 5-FU synergistically upregulate the expression of the pro-apoptotic protein Bax and downregulate the anti-apoptotic protein Bcl-2 [229]. Additionally, the combined treatment significantly inhibits the activation of the PI3K/Akt pathway, affecting cell survival and proliferation [229]. By suppressing thymidylate synthase (TS) or attenuating p-Akt activation, kaempferol enhances the anticancer effects of 5-FU [229].

In addition, kaempferol can reduce glucose uptake and lactic acid production in drug-resistant CRC cells, which can be associated with the attenuation of 5-FU resistance [231,232]. Kaempferol promotes the expression of miR-326 in colon cancer cells, which subsequently inhibits glycolysis by targeting pyruvate kinase M2 isoform (PKM2) 3′-UTR [231]. Similarly, kaempferol also inhibits glycolysis and colon cancer growth by modulating the miR-339-5p-hnRNPA1/PTBP1-PKM2 axis [232]. This reduces the expression of M2-type pyruvate kinase (PKM2) but induces that of PKM1 [232]. Thus, kaempferol attenuates 5-FU resistance in CRC cells by downregulating PKM2-mediated glycolysis. Kaempferol also induce chemo-sensitization to 5-FU in drug-resistant LS174 colon cancer cells by blocking ROS production and modulating the JAK/STAT3, MAPK, PI3K/AKT, and NF-κB signaling pathways [230]. Kaempferol alone or in combination with 5-FU reduces the production of the two angiogenic factors VEGF-A and IL-8 with the downregulation of critical genes involved in drug metabolism, including dihydrofolate reductase (*DHFR*), dihydropyrimidine dehydrogenase (*DPD*), folylpolyglutamate synthetase (*FPGS*), thymidine kinase (*TK*), and thymidylate synthase (*TS*) [230]. Kaempferol combined with 5-FU exerts a synergistic inhibitory effect on cell viability besides inducing cell cycle arrest [230]. The increased activation of MAPK and PI3K/Akt signaling has been observed in oxaliplatin-resistant HT29 and HCT116 cells, which could be mitigated by the anti-cancer effect of kaempferol, resulting in growth inhibition and cell cycle arrest [233]. By targeting serine/threonine kinase RSKs, kaempferol could potentially reduce oxaliplatin-resistant colon cancer cells by suppressing MAPK and PI3K/Akt signaling [233]. The combination of kaempferol and 5-FU/oxaliplatin holds promise as an effective therapeutic strategy for CRC to improve treatment outcomes and minimize adverse effects. However, there are some limitations and considerations related to the development of kaempferol as a chemo-sensitizer in CRC. Kaempferol has poor aqueous solubility, which affects its bioavailability [234]. Formulating it into nano-formulations with biodegradable polymers like chitosan can improve its solubility and absorption [234]. Similarly, more extensive research is still needed to understand the molecular mechanisms and efficacy of kaempferol as well as its interactions with other chemotherapy drugs in CRC.

## 9. Genistein

Genistein is a major isoflavone in soy and soy-based food products, which has been studied for its potential in overcoming drug resistance in CRC [235]. Accumulating evidence demonstrates that genistein as a phytoestrogen exhibits a wide range of anti-cancer effects in CRC, such as apoptosis, cell cycle arrest, anti-angiogenic, anti-metastatic, and anti-inflammatory effects [29,236,237,238]. Genistein can modulate various signaling pathways, including caspases, Bcl-2, ERK1/2, NF-κB, and Wnt/β-catenin, at both transcription and translation levels via the regulation of miRNAs [237,239,240]. For instance, genistein suppresses the proliferation of CRC cells by downregulating miR-95, which in turn inhibits the phosphorylation at T308 within the catalytic domain of Akt, possibly playing a role in apoptosis [241]. Genistein induces apoptosis in colon cancer cells by the reversal of EMT via the Notch1/NF-κB/slug/E-cadherin pathway [242]. Genistein exerts anti-metastatic effects by inhibiting the TTTY18/Akt pathway in CRC cells, which plays a crucial role in cell survival and proliferation [236]. The importance of genistein in CRC is highlighted by its ability to modulate various molecular targets and signal transduction pathways, which may contribute to its potential to counteract drug resistance mechanisms.

The combination of 5-FU and genistein induces apoptosis synergistically in chemoresistant cancer cells through the modulation of AMPK and COX-2 signaling pathways [243]. The combination of 5-FU and genistein synergistically induced apoptosis in chemoresistant HT29 colon cancer cells by modulating the AMPK and COX-2 signaling pathways [243]. The addition of genistein downregulated the COX-2 overexpression and prostaglandin secretion induced by 5-FU treatment in HT-29 colon cancer cells [243]. Further analysis shows that genistein promoted AMPK activation and the upregulation of p53, p21, and Bax, in parallel with the suppression of 5-FU-induced Glut-1 overexpression [243]. 5-FU and genistein also promoted apoptosis in CRC cells by inducing the expression of the DR5 surface protein [243]. AMPK may be a new regulatory molecule of COX-2 expression, which may be involved in the cytotoxicity enhanced by genistein. Furthermore, genistein modulates the anti-cancer effects of cisplatin in HT-29 colon cancer cells by inhibiting cell growth and inducing apoptosis [244]. Interestingly, genistein demonstrates additive effects with cisplatin to suppress cell growth at a concentration of 100 μM but antagonizes the apoptotic effect of cisplatin at 10 μM [244]. Future studies are essential to determine the molecular mechanisms of genistein interacting with cisplatin in colon cancer cells [244].

**Figure 7:**
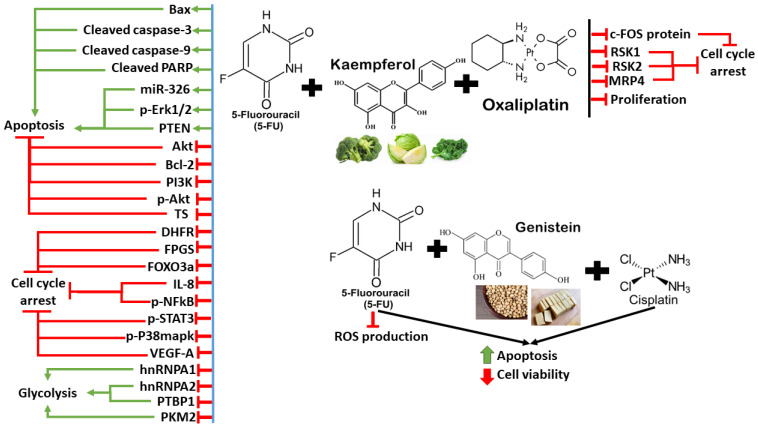
The synergistic mechanisms between kaempferol or genistein and chemotherapy drugs to induce chemo-sensitization in CRC cells. The red symbol represents inhibition, and the green arrow represents upregulation, and the black arrow indicates similar outcomes.

Nevertheless, the possible interactions between genistein and other chemotherapy drugs in CRC are largely unknown. Genistein’s safety profile is generally favorable, but some concerns exist. High doses of genistein may lead to adverse effects, including gastrointestinal disturbances, allergic reactions, and hormonal imbalances. Additionally, the absorption and distribution of genistein in the body may vary between individuals, affecting its therapeutic efficacy. Thus, more research is essential to determine the optimal dosing and administration timing for genistein to be used as chemo-sensitizer in CRC. The synergistic mechanisms between kaempferol or genistein and chemotherapy drugs are illustrated in Figure 7.

## 10. Synergism between Polyphenols and Other Natural Compounds

While polyphenols offer exciting potential in enhancing cancer treatment efficacy by addressing drug resistance in CRC, researchers also delve into the interaction between polyphenols and other natural compounds as a fascinating area of research to overcome chemoresistance. For instance, a combination of curcumin and quercetin shows a synergistic effect in suppressing the proliferation of HCT116 colon cancer cells, although the exact mechanism remains unknown [245]. More recently, Jain et al. have demonstrated that the co-delivery of curcumin and quercetin in shellac nano-capsules enhances the synergistic effects of these two compounds to exhibit antioxidant properties and cytotoxicity against colon cancer cells [246]. As the nanoencapsulation improves the bioavailability of quercetin and curcumin post digestion, both compounds show the highest synergistic efficacy (approximately 80%) at a mass ratio of 4:1 (curcumin/quercetin) [246]. Subsequently, the encapsulated curcumin and quercetin synergistically improve the antioxidant and anti-proliferative activities in HT-29 and HCT-116 CRC cells [246].

Quercetin also synergizes with resveratrol to induce anti-cancer effects in CRC cells by repressing oncogenic miR-27a [247]. The combination of quercetin and resveratrol significantly reduced ROS production and enhanced the antioxidant capacity and apoptotic activity in HT-29 colon cancer cells [247]. Resveratrol and quercetin also synergistically downregulated Sp1, Sp3, and Sp4 and their downstream effector survivin at both transcriptional and translational levels [247]. These Sp factors and survivin are associated with cell proliferation, survival pathways and angiogenesis in cancer cells [62]. The combination of resveratrol and quercetin also inhibited miR-27a to upregulate zinc finger protein ZBTB10 which is a well-known Sp repressor [247]. Thus, the synergism of quercetin and resveratrol modulates the miR-27a-ZBTB10-axis to induce Sp downregulation, leading to anti-cancer activities in CRC cells [247].

Alantolactone (ALT) is a natural olefinic compound found in the traditional Chinese medicinal plant *Inula helenium* L., which has a wide range of biological activities, including anti-inflammatory, antibacterial, and anti-cancer activities [248]. ALT is a selective STAT3 inhibitor that suppresses proliferation and metastasis and promotes apoptosis by modulating the Wnt/β-catenin and MAPKs signaling pathways [248,249]. More importantly, Zhang et al. reported that its co-treatment with quercetin (Q) and alantolactone (A) induced synergistic immunogenic cell death (ICD) in CT26-FL3 colon cancer cells at a molar ratio of 1:4 (Q:A) [250]. The synergistic effects of quercetin and alantolactone can activate anti-tumor immunity by causing cell toxicity, modulating the immune-suppressive tumor microenvironment, and inducing ICD, which could be effective to treat to control aggressive, metastatic, and recurrent CRC that develops resistance against chemotherapeutics [250]. Meanwhile, chrysin is a natural flavone primarily found in honey, propolis, passionflower, and various plants, which has been investigated for its potential roles in cancer therapy [251]. Chrysin exhibits anti-cancer effects by inducing cytotoxicity and stimulating apoptosis, resulting in the inhibition of cancer cell growth and neoplasticity [252,253].

In CRC, chrysin can induce autophagy to suppress the proliferation of CRC cells [254]. More importantly, chrysin can synergize with apigenin to suppress the growth and metastasis of SW480 and HCT116 CRC cells via the attenuation of the P38-MAPK/Akt pathway [255]. The combination of these two polyphenols significantly reduced the proliferation, invasion, and migration ability in both cell lines with the upregulation of apoptosis, suggesting that the synergism between these compounds could become a potential alternative to chemotherapy in CRC treatment [255]. Moreover, curcumin and luteolin can synergize with each other to inhibit tumor progression in CL-188 colon cancer cells by suppressing cell migration, necrosis, and proliferation [256]. Similarly, the combination of curcumin and luteolin also exhibits an inhibitory effect on cancer cell growth in xenograft mice. The synergistic effects between curcumin and luteolin in xenografts were associated with the TGF-β and Notch1 pathways, which are essential to drive tumor progression and metastasis in CRC cells [256].

In addition, the combination between polyphenols and other natural products also shows synergistic effects in CRC xenografts. Trans-pterostilbene (PTS) is a natural polyphenol found in berries, grapes, nuts, and wine, which is also the primary antioxidant component of blueberries [257]. PTS is structurally similar to resveratrol but has higher bioavailability, exhibiting increased lipophilic and oral absorption [257]. PTS has been studied for its potential health benefits, including its antioxidant, anti-inflammatory, and anti-carcinogenic properties [257,258]. The combination of quercetin and PTS has been reported to enhance the efficacy of FOLFOX chemotherapy and/or radiotherapy on HT-29 xenograft growth [259]. Co-treatment with quercetin, PTS, FOLFOX, and radiotherapy significantly reduced the tumor size of HT-29 xenografts associated with long-term survival (>120days) in nude mice models [259]. Gene expression analysis showed that the combination of quercetin and PTS promoted superoxide dismutase 2 via Sp1-dependent transcription regulation and simultaneously suppressed Bcl-2 expression via the inhibition of NF-kB activation [259]. Therefore, the synergistic effects of quercetin and PTS could involve the modulation of superoxide dismutase and Bcl-2 to sensitize CRC xenografts towards chemotherapy and/or radiotherapy.

The synergism between polyphenols may represent another new avenue for the development of novel therapeutics for patients with CRC. This could reduce the use of conventional chemotherapy that often create serious side effects, such as nausea, body weakness, and diarrhea. However, the safety profile and optimal dosage of polyphenols vary among each other and patients with CRC could respond differently towards individual polyphenols. More research is essential to understand the molecular mechanisms underlying the synergism between the polyphenols to induce anti-cancer effects in CRC. The preclinical studies using cell lines and animal studies to investigate the synergistic effects of selected polyphenols in combination with chemotherapeutics and other natural compounds in CRC treatment are summarized in Table 1 and Table 2, respectively.

## 11. Clinical Trials of Polyphenols Combined with Chemotherapy in CRC

While the abovementioned findings about the potential chemo-sensitizing effects of natural polyphenols are promising, it is important to note that most of the research has been conducted in vitro or in animal models. More studies, particularly clinical trials, are needed to fully understand the therapeutic potential and appropriate dosage of natural polyphenols in CRC patients. Clinical trials on polyphenols in combination with chemotherapy or targeted therapy in CRC patients are limited but the outcome is mostly promising. In a randomized phase IIa clinical trial (NCT01490996) that involved 28 patients with stage IV metastatic CRC, the addition of daily oral curcumin consumption (2 g/day) to FOLFOX chemotherapy has been shown to be safe and tolerable, with a significant improvement in overall survival [51]. In another interventional clinical trial (NCT02439385) that recruited 44 colon cancer patients with inoperable metastases after first-line treatment with Bevacizumab/FOLFIRI, the daily consumption of nanostructured lipid particles containing curcumin (100 mg/day) has demonstrated acceptable safety and tolerability with comparable long-term survival rates in combination with chemotherapy or targeted therapy [261]. Gbolahan et al. conducted a phase I clinical trial (NCT01859858) to evaluate the effect of curcumin on the dose-limiting toxicity and pharmacokinetics of irinotecan in participants with solid tumors, including breast, CRC, gastric-esophageal, liver, lung, and pancreatic tumors [262]. The authors concluded that the daily oral consumption of curcumin phosphatidylcholine complex (up to 4 g/day) could be safely administered without impacting the pharmacokinetic and adverse event profile of irinotecan when used in combination [262].

Pintaova et al. also conducted a phase I/II clinical trial (NCT01985763) to assess the safety and tolerability of genistein in combination with FOLFOX or FOLFOX–Bevacizumab for metastatic CRC [263]. They reported that the daily consumption of 60 mg genistein prior to the administration of FOLFOX/FOLFOX-Bevacizumab was safe and tolerable, with a significant improvement in patient response rate and the overall progression-free survival [263]. On the other hand, fisetin is a natural flavonoid mainly found in fruits and vegetables, including apples, strawberries, persimmons, onions, and cucumbers, which has antioxidant, anti-inflammatory, and antiangiogenic properties [264]. In the context of CRC, fisetin induces apoptosis in CRC cells by downregulating Nrf2 and autophagy [265]. Fisetin also enhances the efficacy of ionizing radiation to upregulate tumor suppressor activities and suppress the growth of CRC tumors in xenograft models [266]. A randomized, double blind, placebo-controlled phase I clinical trial (IRCT2015110511288N9) also reported that the addition of oral fisetin (100 mg/day) to oxaliplatin/capecitabine treatment in stage II/III CRC patients significantly reduced the serum levels of C-reactive protein (hs-CRP), interleukin-8 (IL-8) and matrix metalloproteinase-7 (MMP-7) [267]. High serum levels of these proteins are mostly associated with disease progression and systemic inflammation in CRC [268]. Therefore fisetin supplementation could become a novel anti-cancer agent to complement the therapeutic effects of oxaliplatin and capecitabine in patients with CRC, improving inflammatory status and restraining disease progression, which eventually led to better clinical outcome [267]. Meanwhile, most of the abovementioned polyphenols are still in preclinical studies using cell lines and animal models. Given that some polyphenols have limited information on their selectivity, safety profile, long term effects, and possible drug metabolism, there is still a long way to go for the clinical trials using polyphenols as chemo-sensitizers in CRC. Clinical trials evaluating the synergistic effects of selected polyphenols combined with chemotherapy in CRC patients are summarized in Table 3.

## 12. Conclusions, Knowledge Gaps, and Future Research

CRC remains a significant global health challenge, and the emergence of drug resistance poses a formidable obstacle to effective treatment. CRC exhibits remarkable heterogeneity, contributing to both intrinsic and acquired drug resistance. Stem-like cancer cells, with their self-renewal capacity, play a pivotal role in this context. Despite advances in antitumor agents, chemotherapeutic drugs suffer from solubility issues, systemic toxicity, and nonspecific drug delivery and patients with CRC could develop drug resistance, leading to cancer relapse. These drawbacks underscore the need for innovative approaches. In this comprehensive review, we have explored the role of selected polyphenols in overcoming drug resistance with the enhancement of chemosensitivity in CRC. Curcumin, EGCG, quercetin, and resveratrol are regarded as leading candidates to counteract acquired drug resistance. The accumulating evidence from preclinical studies has highlighted the importance of selected polyphenols to synergize with the common chemotherapeutics (5-FU, doxorubicin, irinotecan, and oxaliplatin) and enhance the anti-cancer effects in CRC cells.

Selected polyphenols enhance the chemosensitivity of cancer cells by increasing drug uptake, reducing drug metabolism, and counteracting drug efflux. These polyphenols also modulate autophagy, apoptosis, DNA repair mechanisms, epigenetic regulation, EMT regulation, and ROS production, which contribute towards their synergistic effects with chemotherapy and other natural compounds in overcoming chemoresistance in CRC. Some studies also demonstrate that nanoparticle-based approaches enhance the targeted delivery of selected polyphenols and chemotherapeutics, resulting in better bioavailability and synergistic effects while minimizing systemic toxicity. Rigorous clinical trials are essential to validate the efficacy of selected polyphenols combined with existing chemotherapeutic agents or targeted therapies in CRC treatment. These studies should consider patient heterogeneity and long-term outcomes to improve treatment efficacy and minimize side effects. In summary, selected polyphenols hold promise as adjuvants in CRC therapy. By addressing current challenges, bridging knowledge gaps, and focusing on future research avenues, we can harness their potential to overcome drug resistance and improve patient outcomes.

## Figures and Tables

**Table 1:** In vitro studies on the synergistic effects of selected polyphenols in combination with chemotherapeutics and other natural compounds in CRC treatment.

Polyphenol	Combination with	Cell Line Model	Main Outcome	Reference
Curcumin	5-FU	HCT116 and HCT116 + ch3 parental cells; 5FU-resistant clones (HCT116R, HCT116 + ch3R)	↓ Colonosphere formation, cell growth ↑ Apoptosis ↓ ALDH1, CD44, CD133	[64]
	5-FU	HCT116 and SW480 parental cells; 5FU-resistant clones (HCT116-5FUR, SW480-5FUR)	↓ EMT, proliferation ↑ Apoptosis, cell cycle arrest ↑ miR-200b, miR-200c, miR-141, miR-429 and miR-101 ↓ EZH2, BMI1, SUZ12 and Ring1B	[26]
	5-FU	5FU-resistant HCT8 cells	↑ Apoptosis, cell cycle arrest (G0/G1) ↓ HSP27, P-gp	[74]
	5-FU	5FU-resistant HCT116 cells	↓ EMT, proliferation, WNT signaling ↑ Apoptosis, cell cycle arrest (G0/G1) ↓ NKD2, TET1	[73]
	Cisplatin	HCT8 parental cells and HCT8/DDP-resistant cells	↓ Cell viability ↑ Apoptosis ↓LncRNA KCNQ1OT1, Bcl2 ↑ miR-497	[87]
	Cisplatin	HT-29 and LoVo cells; cisplatin-resistant HT-29 cells	↓ Proliferation ↑ Apoptosis ↑ miR-137 ↓ Glutaminase	[63]
	Dasatinib	HCT116 and HT-29 parental cells; resistant cells (CR-HCT116 and CR-HT-29)	↓ Cell growth, colonosphere size. and extracellular matrix invasion ↓CD133, CD44, CD166, and ALDH1	[65]
	Dasatinib	HCT-116 p53w), HT-29, HCT-116 p53^−/−^, and SW-620 cells	↓ Cell growth, colony formation and metastasis3 ↓ BcL_xL_, COX-2, EGFRs, IGF-1R c-Src signaling, p-Akt, p-Erk and NF-κB	[107]
	Doxorubicin	Doxorubicin-resistant HT-29 cells	↓ Colony formation, proliferation ↓ COX-2, LDH, MDR1, P-gp	[93]
	FOLFOX	Chemo-surviving HCT-116 and HT-29 cells	↓ Cell viability ↓ AKT, COX-2, cyclin-D1, EGFR, HER-2, IGF-1R	[95]
	Irinotecan (CPT-11)	LoVo parental cells and LoVo/CPT-11-resistant cells	↓ Cell growth and tumor sphere formation ↓ CD24, CD44, CD133 and EpCAM ↑ Apoptosis	[89]
	Irinotecan (CPT-11)	LoVo parental cells and LoVo/CPT-11R-resistant cells	↓ Proliferation ↑ Apoptosis ↑ E-cadherin ↓ Vimentin and N-cadherin	[90]
	Irinotecan	LoVo and HT-29 cells	↓ Cell viability ↑ Apoptosis, cell cycle arrest, ER stress, ROS generation	[88]
	Oxaliplatin	Oxaliplatin-resistant HCT116 p53wt and p53−/− cells	↓ Proliferative capacity ↓ ALDH1, Notch1, survivin ↑ p21, p53	[77]
	Oxaliplatin	HT29, LoVo, and DLD1 parental cells. Oxaliplatin-resistant sublines HTOXAR3, LoVOXAR3 and DLDOXAR3,	↓ Proliferation and colony formation ↓ CXCL1, CXCL8, and CXCL2 ↓ Akt/NF-κB pathway	[82]
	Oxaliplatin	HCT116 parental cells and HCT116/OXA	↓ Cell growth and EMT ↓ Bcl-2, p-p65 p-Smad2, p-Smad3, and N-cadherin ↑ Cleaved caspase-3 and E-cadherin	[84]
Curcuminoids	5-FU	HT-29 and SW480 cells	↓ Cell growth ↑ ROS production ↓ MDR1, miR-27a, Sp1, Sp3, and Sp4 ↑ ZBTB10	[75]
Resveratrol	5-FU	HCT-116 parental cells and 5-FU-resistant HCT-116R	↓ Cell viability, colony formation, invasion, migration, and proliferation ↓ ALDH1, β1-integrin CD44, CD133, HIF-1α, NF-kB, and VEGF	[130]
	5-FU	HCT116 and SW480 parental cells; 5-FU-chemoresistant derived clones (HCT116R and SW480R)	↓ EMT and proliferation ↑ Claudin-2, desmosomes, and E-cadherin ↓ Caspase-3, IκBα kinase, IκBα, MMP-9, NF-κB, vimentin, and slug	[25]
	5-FU	DLD-1, SW480 and COLO201 parental cells; 5-FU-resistant (DLD-1/5FU)	↓ Cell viability and proliferation ↑ Apoptosis ↑ miR-34a ↓ MAPK/Erk1/2 signaling and PI3K/Akt signaling ↓ E2F3/Sirt1	[42]
	5-FU	DLD-1 and HCT116 cells	↓ Cell viability and EMT ↑ Apoptosis, cell cycle arrest ↓ Akt, NF-κB, p-NFκB, p-STAT3, and STAT3	[117]
	5-FU	HCT116 parental cells; 5-FU-chemoresistant derived clone (HCT116R)	↓ Stemness, EMT, proliferation and TNF-β induced chemoresistance ↑ Cleaved caspase-3, E-cadherin ↓ ALDH1, CD133, CD44, CXCR4, MMP-9, NF-κB, vimentin and slug	[118]
	5-FU	HT-29 and SW620 cells	↓ Cell viability ↑ Apoptosis, lipid peroxide accumulation, and ROS production ↓ Akt and STAT3	[119]
	Doxorubicin	Caco-2 cells	↓ Cell viability ↑ Apoptosis ↑ Caspase-3, caspase-8, and caspase-6/9 ↓ BCRP, CYP3A4 GST, hPXR, MDR1, and MRP1	[125]
	Doxorubicin	HCT116 and HT-29 cells	↓ Cell viability ↑ Apoptosis, cell cycle arrest ↑ p53 and Bax ↓ Bcl-xL and P-gp	[140]
	Oxaliplatin	HCT116 and HT-29 cells	↓ Cell viability, invasion, migration, and proliferation ↑ Apoptosis ↑ miR-34c ↓ PI3K/Akt	[139]
	Oxaliplatin	Caco-2 cells	↓ Cell viability ↑ Apoptosis, DNA fragmentation, secondary necrosis, and alteration in cytokine profile ↑ Cleaved caspase-3, cleaved PARP, IL-8, IL-10 and TNF-α	[120]
EGCG	5-FU	DLD-1 and HCT116	↓ Cell viability ↑ Apoptosis, DNA damage ↑ Cleaved caspase-3 and PARP, miR-155-5p, and NF-κB ↓ Bcl-2, GRP78, MDR1	[157]
	5-FU	HCT116 and SW480 parental cells; 5FU-resistant clones (5FUR-HCT116 and 5FUR-SW480)	↓ Cell viability, proliferation, and spheroid-forming capacity. ↑ Apoptosis, cell cycle arrest, ↑ miR-34a, miR-145, and miR-200c ↓ Bmi1, Ezh2, Notch1 and Suz12.	[156]
	Cisplatin	DLD-1 and HT-29 cells	↓ Proliferation ↑ Apoptosis and autophagy ↑ Acidic vesicular organelles, autophagosome and LC3-II protein.	[164]
	Irinotecan	HCT116 and RKO cells	↓ Cell viability, invasion, and migration ↑ Apoptosis, autophagy, cell cycle arrest, and DNA damage ↑ ATM, p-ATM, p-RB and γ-H2AX ↓ CDK4, Cyclin B1 and Cyclin D1 and TOP1	[161]
	Oxaliplatin	DLD-1 and HT-29 cells	↓ Proliferation ↑ Apoptosis and autophagy ↑ Acidic vesicular organelles, autophagosome, and LC3-II protein.	[164]
Quercetin	5-FU	CO115 p53wt, HCT15 p53mt, HCT116 p53wt and HCT116 p53mt cells	↓ Cell viability and proliferation ↑ Apoptosis ↓ Bcl-2 ↑ Cleaved caspase-3/9 and PARP, p53	[173]
	5-FU	HCT116 parental cells and 5-FU-resistant HCT116 cells (HCT116-R)	↓ Cell viability, proliferation, and ROS production ↑ Apoptosis ↓ CAT, GPx, GR, HO-1, Nrf2, SOD-1, and p-IKBα.	[178]
	5-FU	HT-29 cells	↓ Cell viability ↑ Apoptosis ↓ Akt, Bcl-2, mTOR, VEGF ↑ Bax, p38 MAPK, p53, and PTEN	[187]
	5-FU	Resistin-treated DLD-1 and HCT116 cells	↓ Cell viability ↑ Apoptosis ↓ NLRP3 and p-ERK	[189]
	Alantolactone	CT26-FL3 cells	↑ Immunogenic cell death	[250]
	Curcumin (co-delivery in shellac nanocapsules)	HT-29 and HCT116 cells	↓ Proliferation ↑ Antioxidant capacity	[246]
	Doxorubicin	HT-29 cells	↓ Cell viability, proliferation, and stemness ↑ Apoptosis and cell cycle arrest	[191]
	Doxorubicin	P-gp-overexpressed SW620/Ad300 cells	↓ D-glutamine and D-glutamate metabolism ↓ Proliferation, ↑ Apoptosis ↓ P-gp and SLC1A5	[190]
	FOLFOX + Trans-pterostilbene + radiotherapy	HT-29 cells	↓ Cell viability ↑ SOD2 ↓ Bcl-2, IκBα, p-IκBα, NF-κB,	[259]
	Oxaliplatin	HCT116 cells	↓ Cell viability, glutathione reductase activity, and intracellular glutathione ↑ ROS production	[188]
	Resveratrol	HT-29 cells	↓ ROS production ↑ Apoptosis and antioxidant capacity ↑ Cleaved caspase-3 and PARP, ZBTB10 ↓ miR-27a, Sp1, Sp3, Sp4, and survivin	[247]
Kaempferol	5-FU	LS174 parental cells and 5-FU-resistant LS174-R cells	↓ Cell viability and ROS production ↑ Apoptosis, cell cycle arrest, ↑ Cleaved caspase-3, caspase-9 and PARP, p-Erk1/2 ↓ DHFR, FPGS, FOXO3a, IL-8, p-Akt, p-NFκB, p-STAT3, p-p38MAPK, VEGF-A, TK, and TS	[230]
	5-FU	HCT-8 or HCT-116	↓ Cell viability and proliferation ↑ Apoptosis ↑ Bax and PTEN ↓ Akt, Bcl-2, PI3K, p-AKT and TS	[229]
	5-FU	HCT-8 parental cells and 5-FU-resistant HCT-8R cells	↓ Cell viability, glycolysis, and proliferation ↑ Apoptosis ↑ miR-326 ↓ hnRNPA1, hnRNPA2, PTBP1 and PKM2	[231]
	Oxaliplatin	Oxaliplatin-sensitive HCT116 (HCT116-Ox^S^) and HT-29 (HT29-Ox^S^) parental cells; Oxaliplatin-resistant HCT116 (HCT116-Ox^R^) and HT-29 (HT29-Ox^R^) cells	↓ Proliferation ↑ Cell cycle arrest ↓ c-Fos protein, RSK1, and RSK2	[233]
Genistein	5-FU	HT-29 cells	↓ Cell viability and ROS production ↑ Apoptosis ↓ COX-2, Glut1 ↑ AMPK, PARP, p21, p53	[243]
	Cisplatin	HT-29 cells	↓ Cell viability ↑ Apoptosis	[244]
Apigenin	5-FU	HCT116 and HT-29 cells	↓ Cell viability and proliferation ↑Apoptosis, cell cycle arrest, Ca^2+^ accumulation, mitochondrial membrane disruption, and ROS production ↓ TS	[196]
	5-FU (dual-drug-loaded liposomal nanocarrier)	HCT-15 and HT-29 cells	↓ Cell viability, colony formation and proliferation ↑ Apoptosis, angiogenesis, cell cycle arrest mitochondrial membrane destabilization and ROS production ↓ COX-2, HIF-1α and p-mTOR ↑ p-AMPK	[197]
	Chrysin	HCT116 and SW480 cells	↓ Colony formation, invasion, and migration ↑ Apoptosis ↓ p-P38 and p-AKT	[255]
	Cisplatin	Cisplatin-resistant HT-29 cells	↓ Cell viability ↑ Apoptosis and autophagy ↓ Bcl-2, p-mTOR, p-PI3K, and p-AKT ↑ Bax	[200]
	Irinotecan	HT-29 and HRT-18 cells	↑ CD26, DPPIV and eADA	[199]
Luteolin	5-FU	HT-29 cells	↓ Cell viability ↑ Apoptosis ↓ Akt, Bcl-2, mTOR, VEGF ↑ Bax, p38 MAPK, p53, and PTEN	[187]
	Baicalein	LoVo parental cells and doxorubicin-resistant subline (LoVo/Dx)	↓ Cell viability and proliferation ↑ Apoptosis and DNA fragmentation ↓ PCNA ↑ Caspase-3 activity	[260]
	Curcumin	CL-188 and DLD-1 cells	↓ Cell proliferation and migration ↓ Notch1 and TGF-β	[256]
	Oxaliplatin	SW480 cells	↓ Cell viability ↑ Apoptosis and oxaliplatin uptake ↑ PDZK1, PDZK2, and OCTN2	[219]
	Oxaliplatin	HCT116 and SW620 parental cells; oxaliplatin-resistant lines (HCT116-OX and SW620-OX)	↓ Cell viability ↓ Nrf2 and NQO1	[211]
	Oxaliplatin	HCT116 cells (p53^+/+^ and p53^−/−^)	↓ Cell viability, colony formation, and cell cycle arrest ↑ Apoptosis ↑ Akt, cleaved PARP, p53, Nrf2/ARE/HO-1 axis.	[212]

↑ Upregulation; ↓ Downregulation.

**Table 2:** Xenograft animal studies on the synergistic effects of selected polyphenols in combination with chemotherapeutics and other natural compounds in CRC treatment.

Polyphenol	Combination with	Animal Model	Main Outcome	Reference
Curcumin	5-FU	HCT116-5FUR xenografts in athymic nude mice	↑ 5-FU sensitivity ↑ miR-200c	[26]
	Oxaliplatin	HCT116 p53wt xenografts in nude mice	↓ Tumor volume ↓ Ki67, Notch1 ↑ Cleaved caspase-3	[77]
Resveratrol	Oxaliplatin	HCT116 xenograft in BALB/c nude mouse	↓ Tumor size ↑ miR-34c ↓ IL-6, KITLG	[139]
Quercetin	FOLFOX + Trans-pterostilbene + radiotherapy	HT-29 xenograft in nu/nu nude mice	↓ Tumor volume ↑ Bak, Bax, Bad, Bid, CAT, GPx, GR, SOD2, TrxR-1 ↓ Bcl-2	[259]
	Oxaliplatin	HCT116 xenograft in male BALB/c nude mice	↓ Tumor growth ↑ Bax/Bcl-2, cytochrome C, cleaved PARP ↓ Tumor size, ABCG2, and MRP4	[188]
Apigenin	5-FU (dual-drug-loaded liposomal nanocarrier)	HT-29 xenograft in nude mice	↑ In vivo tumor regression ↓ In vivo cellular proliferation and vascularization	[197]
	Cisplatin	HT-29 xenograft in BALB/c nude mice	↓ Tumor growth	[200]
Luteolin	Curcumin	CL-188 xenograft in male nude BALB/c mice	↓ Tumor growth ↑ Necrosis ↓ Notch1 and TGF-β	[256]
	Oxaliplatin	HCT116 xenografts in BALB/c nude mice	↓ Tumor size ↑ Apoptosis ↑ Cleaved PARP and p53 ↓ AMPK ↑ Akt, Nrf2/ARE/HO-1 axis.	[214]

↑ Upregulation; ↓ Downregulation.

**Table 3:** Clinical trials evaluating the synergistic effects of selected polyphenols combined with chemotherapy in CRC patients.

Polyphenol (Dose)	Subjects/Study Type	Experimental Group	Control Group	Main Outcome	Clinical Trial Identifier and Reference
Curcumin (C3 complexed, 2 g/day)	Stage IV mCRC pts/Phase IIa randomized controlled	FOLFOX + curcumin (CUFOX, *n* = 18)	FOLFOX (*n* = 9)	↑ PFS ↑ OS = QOL ↓ Neuropathy scores (trend)	NCT01490996 [51]
Curcumin (nanostructured lipid particles, 100 mg twice/day)	Stage IV mCRC pts/Interventional Phase II	FOLFIRI/Bevacizumab + curcumin (*n* = 44)	None	Comparable long-term survival outcomes with acceptable toxicity outcomes	NCT02439385 [261]
Curcumin phosphatidylcholine complex (1, 2, 3, and 4 g once per day)	CRC pts/Phase 1 pilot study	Curcumin complex + irinotecan (*n* = 23)	None	Up to 4 g of curcumin complex could be safely administered with irinotecan	NCT01859858 [262]
Genistein (60 mg/day)	Stage IV mCRC pts/Interventional Phase I/II pilot	FOLFOX + genistein (n = 10) FOLFOX/Bevacizumab + genistein (*n* = 10)	None	↑ RR ↑ PFS	NCT01985763 [263]
Fisetin (100 mg/day)	Stage II/III CRC pts/randomized placebo-controlled Phase I pilot	Oxaliplatin infusion/oral capecitabine + fisetin (*n* = 18)	Oxaliplatin infusion/oral capecitabine + placebo (corn starch, *n* = 19)	↓ Serum CRP, IL-8, MMP-7	IRCT2015110511288N9 [267]

C-reactive protein (CRP), overall survival (OS), progression-free survival (PFS), quality of life (QOL), and response rate (RR). ↑ Upregulation; ↓ Downregulation.
